# The heterogeneity and complexity of *Cannabis* extracts as antitumor agents

**DOI:** 10.18632/oncotarget.26983

**Published:** 2019-06-25

**Authors:** Liran Baram, Ella Peled, Paula Berman, Ben Yellin, Elazar Besser, Maya Benami, Igal Louria-Hayon, Gil M. Lewitus, David Meiri

**Affiliations:** ^1^The Laboratory of Cancer Biology and Cannabinoid Research, Department of Biology, Technion - Israel Institute of Technology, Haifa, Israel

**Keywords:** cancer, *Cannabis*, cannabinoids, (-)-Δ^9^-*trans*-tetrahydrocannabinol (Δ^9^-THC), antitumor

## Abstract

The *Cannabis* plant contains over 100 phytocannabinoids and hundreds of other components. The biological effects and interplay of these *Cannabis* compounds are not fully understood and yet influence the plant’s therapeutic effects. Here we assessed the antitumor effects of whole *Cannabis* extracts, which contained significant amounts of differing phytocannabinoids, on different cancer lines from various tumor origins. We first utilized our novel electrospray ionization liquid chromatography mass spectrometry method to analyze the phytocannabinoid contents of 124 *Cannabis* extracts. We then monitored the effects of 12 chosen different *Cannabis* extracts on 12 cancer cell lines. Our results show that specific *Cannabis* extracts impaired the survival and proliferation of cancer cell lines as well as induced apoptosis. Our findings showed that pure (-)-Δ^9^-*trans*-tetrahydrocannabinol (Δ^9^-THC) did not produce the same effects on these cell lines as the whole *Cannabis* extracts. Furthermore, *Cannabis* extracts with similar amounts of Δ^9^-THC produced significantly different effects on the survival of specific cancer cells. In addition, we demonstrated that specific *Cannabis* extracts may selectively and differentially affect cancer cells and differing cancer cell lines from the same organ origin. We also found that cannabimimetic receptors were differentially expressed among various cancer cell lines and suggest that this receptor diversity may contribute to the heterogeneous effects produced by the differing *Cannabis* extracts on each cell line. Our overall findings indicate that the effect of a *Cannabis* extract on a specific cancer cell line relies on the extract’s composition as well as on certain characteristics of the targeted cells.

## INTRODUCTION

Of the over 500 different compounds present in the *Cannabis* plant, currently more than 120 have been identified as phytocannabinoids, the unique bioactive compounds of the *Cannabis* plant [1]. The two most well-known and heavily researched of these compounds are (-)-Δ^9^-*trans*-tetrahydrocannabinol (Δ^9^-THC), a principal psychotropic ingredient in the plant [2, 3], and cannabidiol (CBD) [4]. However, many other phytocannabinoids which found in *Cannabis* at varying levels, are less studied but still presumed to have pharmacological properties [5].

Many of the phytocannabinoids found in *Cannabis* affect the endocannabinoid system (ECS), a principal endogenous signaling system that appeared early in evolution and has important regulatory functions throughout the body [6–8]. This system consists of two main cannabinoid receptors (CB1 and CB2). More recently, additional cannabinoids have been shown to bind to other non-CB1, non-CB2 receptors, including the G protein-coupled receptor 55 (GPR55) [9] or the transient receptor potential (TRP) channels (TRPV1, TRPV2, TRPA1, TRPM8) [10]. Following the terminology by Leishman *et al.*, 2015 [11], receptors which interact with cannabinoids are referred to as “cannabimimetic receptors”. Some of these receptors were found to be upregulated in cancer, although conflicting reports exist regarding the role of the ECS in tumor generation and progression [12–17].

In the last decade, accumulating evidence has indicated that phytocannabinoids might have antitumor properties. A number of *in vitro* and *in vivo* studies have demonstrated the effects of phytocannabinoids on tumor progression by interrupting several characteristic features of cancer. These studies suggest that specific cannabinoids such as Δ^9^-THC and CBD induce apoptosis and inhibit proliferation in various cancer cell lines at concentrations ranging from 5 to 65 µM [18–25].

Hundreds of different *Cannabis* species and hybrids exist worldwide, which vary significantly in their phytocannabinoid compositions. Certain combinations and concentrations of phytocannabinoids and their interplay may determine its medicinal effects and adverse side effects [26, 27]. In 2015, Armstrong et al. [21] revealed that combinations of CBD with Δ^9^-THC were more effective in reducing melanoma cell viability than applying Δ^9^-THC alone. Nabissi et al. (2016) showed that a combination of CBD and Δ^9^-THC induced multiple myeloma cell death, while administration of pure Δ^9^-THC or CBD alone did not [28]. In a recently published study, Blasco-Benito et al. (2018) demonstrated the advantage of using a whole *Cannabis* extract over pure Δ^9^-THC by comparing their antitumor effects on breast adenocarcinoma cell lines [29]. These studies suggest a synergistic effect of various *Cannabis* compounds and therefore, it is of the utmost importance to study the antitumor effects of whole *Cannabis* extracts.

In this research we attempt to characterize the antitumor effects of 12 whole *Cannabis* extracts on 12 different cancer cell lines sourced from different tumor origins. We evaluate the effects of these *Cannabis* extracts to determine whether whole *Cannabis* preparations with specific phytocannabinoid profiles could be advantageous as therapy for certain cancer sub-types.

## RESULTS

### The heterogeneous composition of *Cannabis* extracts

**Figure 1:**
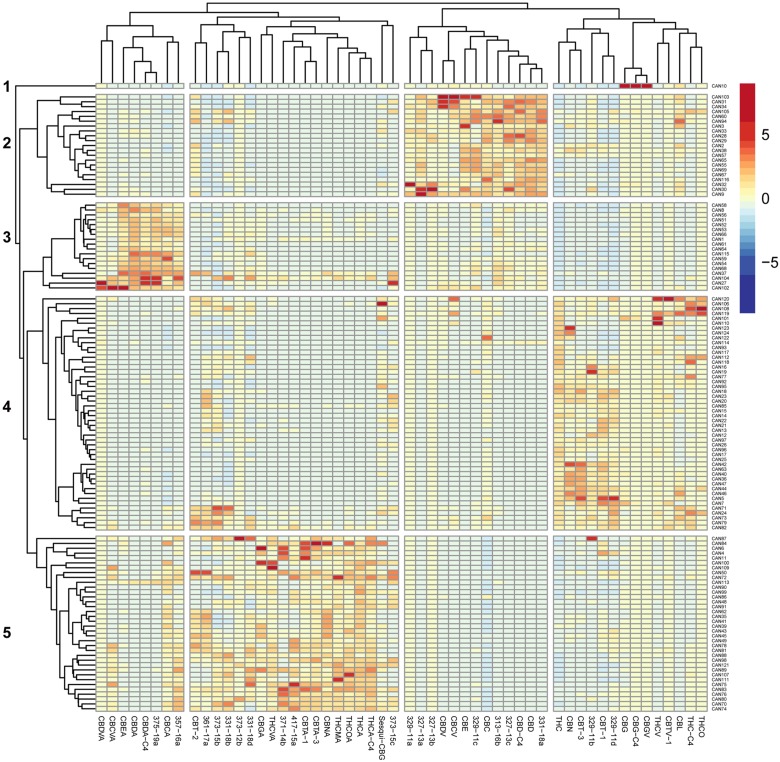
Heat map of unsupervised hierarchical clustering of the cannabinoid profile of 124 *Cannabis* extracts. The matrix of the ESI-LC/MS phytocannabinoid analysis Z-scores representing the set of associations was scaled by column to range from -8 to 8. Negative values (dark blue) indicate that the extract contained very low levels of the phytocannabinoid, and positive values (red) indicate that this extract was composed of high levels of the phytocannabinoid. Dendrograms indicating the clustering relationships are shown to the left and above the heat map. The 124 extracts segregate into five major clusters comprised of phytocannabinoids that associate with: (1) larger amounts of CBG-type; (2) larger amounts of CBD-type.; (3) larger amounts of CBDA-type; (4) larger amounts of Δ^9^-THC-type; (5) larger amounts of Δ^9^-THCA-type.

In order to comprehensively quantify phytocannabinoids in the 124 natural and decarboxylated *Cannabis* extracts, we applied an electrospray ionization liquid chromatography mass spectrometry (ESI-LC/MS) method recently developed in our lab [30]. Overall, 89 phytocannabinoids were observed in these extracts, of which 54 phytocannabinoids are presented in the heat-map in Figure 1. Criteria for a phytocannabinoid’s inclusion in the analysis was its detection in at least three extracts and a minimum concentration of 0.1 % w/w in any of the studied extracts. According to Figure 1, significant differences in phytocannabinoid compositions exist among the 124 *Cannabis* extracts. Hierarchical clustering of the corresponding *z*-score matrix of association showed five major clusters (1-5) characterized by patterns of phytocannabinoid compositions. According to this hierarchical clustering, it is apparent that minor constituents can also significantly contribute to the variance among *Cannabis* extracts, as previously suggested by Berman et al. (2018) [30].

Based on the extract profiling results, we selected 12 representative *Cannabis* extracts (CAN1-CAN12) from these five clusters which varied significantly in their phytocannabinoid compositions (Figure 1, Supplementary Figure 1). For example, CAN2, CAN3 and CAN9 contained more CBD- and cannabichromene (CBC)-type phytocannabinoids and degradation products (cluster 2), while CAN1 and CAN8 exhibited large concentrations of cannabidiolic acid (CBDA)-type phytocannabinoids and degradation products (cannabielsoic acid, CBEA-type) and were categorized in cluster 3. Similarly, CAN5, CAN7 and CAN12 contained relatively higher concentrations (>35%) of Δ^9^-THC-type phytocannabinoids and were categorized in cluster 4, while CAN4, CAN6 and CAN11 all consisted of relatively high contents of (-)-Δ^9^-*trans*-tetrahydrocannabinolic acid (Δ^9^-THCA)-type phytocannabinoids and degradation products (cannabinolic acid, CBNA- and (±)-*trans*/*cis*-cannabitriolic acid, CBTA-types) and were therefore categorized in cluster 5. CAN10 had the highest concentration of cannabigerol (CBG)-type phytocannabinoids compared to all other samples, and therefore was individually categorized as cluster 1. Interestingly, some of the additional phytocannabinoids identified in our previous publication [30], whose absolute chemical structures could not be determined with certainty at this point, were also observed and clustered in the different groups. For example, 329-11a and 329-11c, 327-13a to 327-13c, 313-16b and 331-18a were identified in decarboxylated CBD-rich extracts, whereas 329-11b and 329-11d were identified in Δ^9^-THC-rich extracts. Specific phytocannabinoids can also be observed clustered with the acid-type phytocannabinoids. These agree with the neutral-acid pairing suggested in [30] according to the MS/MS fragmentation spectra (for example, 357-16a is the acid form of 313-16b, and both are most highly expressed in CBD-rich extracts). This suggests of mutual biosynthetic and/or degradation pathways with the major phytocannabinoids. Interestingly, 331-18b and 331-18d, are highly expressed in Δ^9^-THCA-rich extracts (as well as in all other samples), although according to their MS/MS spectra they are neutral phytocannabinoids.

### Effect of *Cannabis* on cancer cell line survival

**Figure 2:**
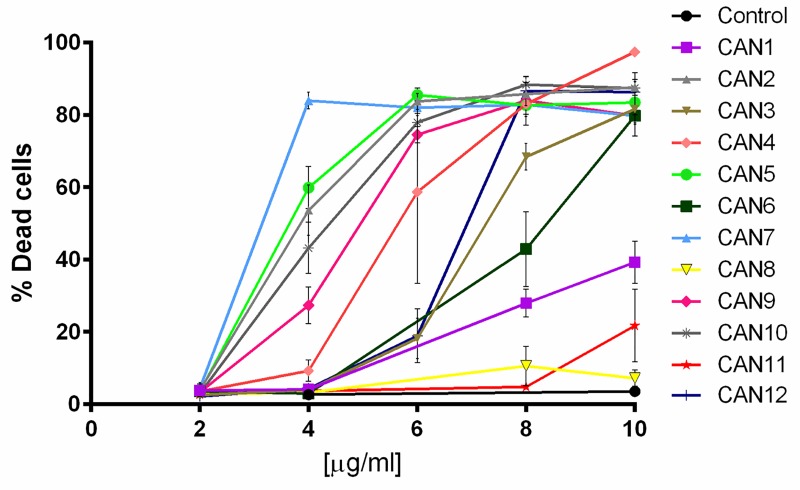
The effect of various *Cannabis* extracts on the survival of cancer cells. A dose-response curve of A549 cells after 24 h incubation with or without (control) 2-10 μg/ml of CAN1-CAN12 calculated from at least 5 independent experiments.

We then examined the effects of the 12 chosen *Cannabis* extracts (CAN1 – CAN12) on the survival of 12 well-characterized cancer cell lines. Cells were treated with increasing concentrations (2-10 µg/ml) of the *Cannabis* extracts for 24 h, and cell survival was monitored using Hoechst and PI staining. The effects of these 12 differing *Cannabis* extracts on the survival of A549 cells, a lung carcinoma cell line, is shown in Figure 2. As shown, CAN2, CAN3, CAN4, CAN5, CAN6, CAN7, CAN9, CAN10 and CAN12 affected A549 cell survival in a dose-dependent manner as opposed to CAN8 and CAN11 which did not significantly affect cell viability.

**Figure 3:**
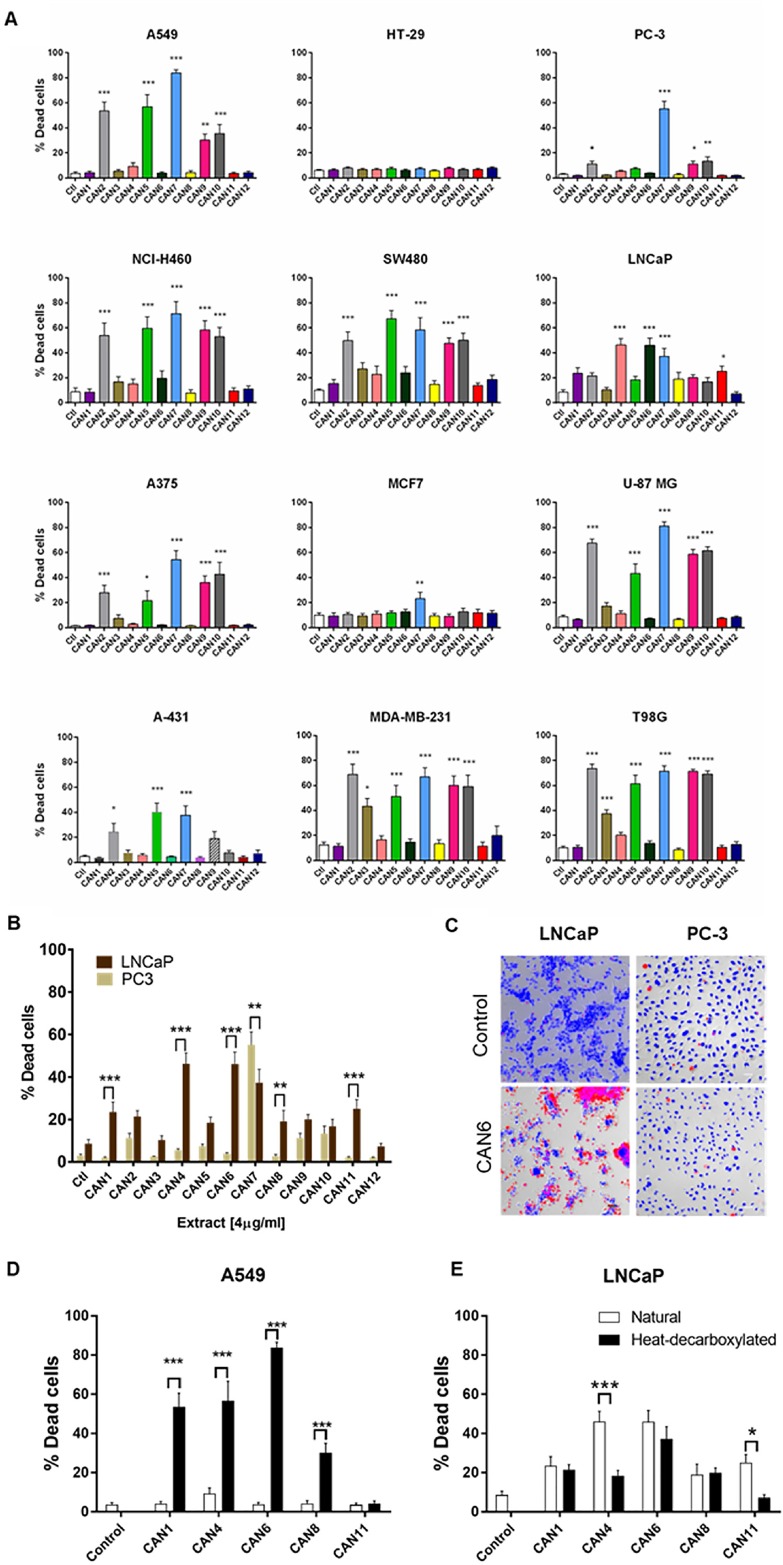
Differential effect of different *Cannabis* extracts on the survival of various cancer cells. Cancer cell lines of various tumor origins were treated with 4 µg/ml of 12 different *Cannabis* extracts for 24 h. Data are reported as mean ± SE of % dead cells out of total cells (N=7). **(A)** The effect of different *Cannabis* extracts on cell lines A549, NCI-H460, A375, A-431, SW480, HT-29, MCF7, MDA-MB-231, LNCaP, PC-3, U-87 MG and T98G. Asterisks represent statistically significant differences compared to control (*P < 0.05, **P < 0.005, ***P < 0.0005; one-way ANOVA). **(B)** A comparison between the effect of *Cannabis* extracts on PC-3 and LNCaP prostate carcinoma cell lines. Asterisks indicate statistically significant differences between LNCaP and PC-3 cell lines (*P < 0.05, **P <0.005, *P < 0.0005; two-way ANOVA with Bonferroni's post hoc multiple comparisons test) **(C)** Representative fluorescent images overlaid onto transmitted light images of LNCaP and PC-3 prostate cancer cells treated with or without (control) 4 µg/ml of CAN6 (blue- Hoechst- all cells, red- PI- dead cells). **(D-E)** A comparison between the effect of neutral (white columns) and heat-decarboxylated (black columns) phytocannabinoid contents of *Cannabis* extracts on A549 and LNCaP cells. Asterisks indicate statistically significant differences between extracts (*P < 0.05, **P < 0.005, ***P < 0.0005; two-way ANOVA with Bonferroni's post hoc multiple comparisons test).

Each extract’s effects on cell survival was then assessed on 12 human cancer cell lines (Supplementary Table 1 and Figure 3A demonstrating the effects of 4 µg/ml of each extract on these cells). Excluding its effects on the HT-29 cell line, CAN7 on average created the most potent cytotoxic effects on the selected cancer cell lines, with IC50 values ranging from 3.06 to 5.74 µg/ml. With IC50 values above 10 µg/ml, CAN1, CAN8 and CAN11 were the least potent extracts on all the cancer cell lines excluding the LNCaP cell line (Figure 3, Supplementary Table 1).

These varying effects of the *Cannabis* extracts on the survival of cancer cell lines appeared in most of the cell lines tested (Figure 3A). Interestingly, differing cancer cell lines from the same organ origin were affected differently by the same *Cannabis* extracts. For example, in comparison to the PC-3 prostate cancer cell line, which was more susceptible to CAN7, the LNCaP prostate cancer cell line was more sensitive to the effects produced by the CAN1, CAN4, CAN6, CAN8 and CAN11 extracts (Figure 3A-3C).

Overall, CAN2, CAN5, CAN7, CAN9 and CAN10 extracts were found to be the most potent in affecting cancer cell survival above all other tested extracts (Figure 3A, Supplementary Table 1). The common feature that these extracts shared was that they contained a high content (≥50% w/w) of phytocannabinoids in their decarboxylated form. However, this phenomenon was not uniformly produced in all cells tested, as presented in Figure 3D and 3E. The extracts comprising mainly of phytocannabinoids in their decarboxylated forms were significantly more potent in affecting cell survival on the A549 cell line (Figure 3D). However, *Cannabis* extracts containing more phytocannabinoids in their natural acid forms were more potent in reducing the survival of the LNCaP prostate carcinoma cell line (Figure 3E). These results emphasize the selective nature of *Cannabis* extracts to affect the survival a certain cancer cell line.

### Anti-proliferative and proapoptotic effects of *Cannabis* extracts

**Figure 4:**
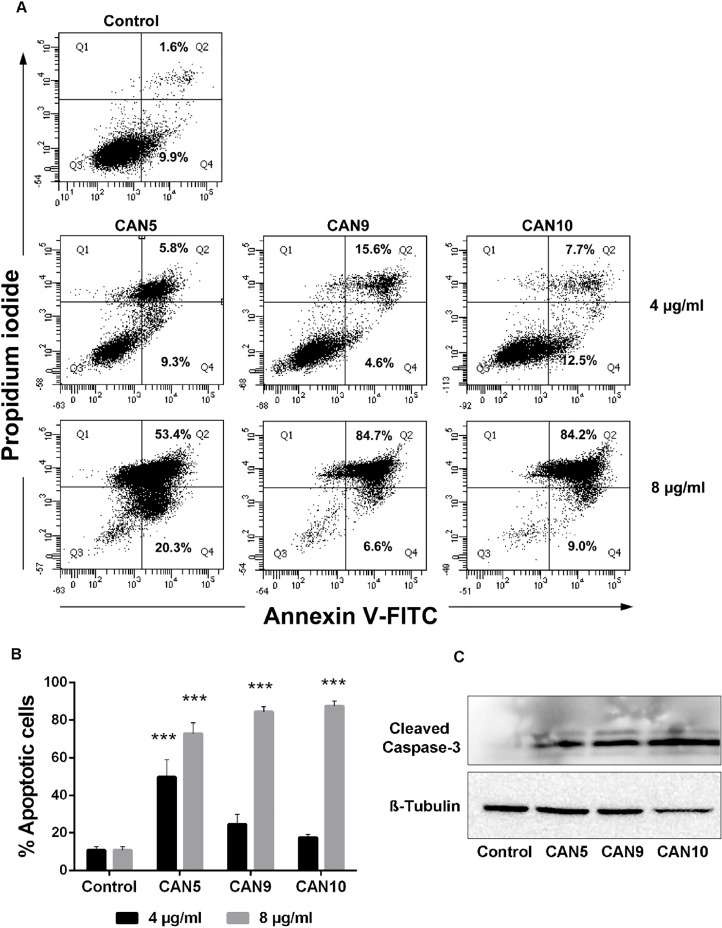
Proapoptotic effect of *Cannabis* extracts on cancer cells. A549 cells were incubated for 24 hours with 4 or 8 μg/mL CAN5, CAN9, CAN10 extracts or with DMSO (control). Apoptosis (early and late) was assessed by Annexin V/PI staining using flow cytometry. Results were calculated as % of positive annexin V-FITC cells out of total cells counted. **(A)** Representative dot plots of cells treated with 4 or 8 μg/ml CAN5, CAN9 and CAN10 extracts. **(B)** Bar chart of total apoptosis following incubation with extracts. Data are presented as mean ± SE (N=5). Asterisks indicate statistically significant differences compared to control (***P < 0.0001; two-way ANOVA with Bonferroni's post hoc multiple comparisons test). **(C)** Cells were lysed and resolved on 15% SDS-PAGE followed by western blotting with anti-cleaved caspase 3 and Anti β-Tubulin antibodies.

In order to verify the cause of the reduction in cell survival following *Cannabis* exposure, we examined the abilities of various *Cannabis* extracts to induce cell death via apoptosis. A549 cells were treated with three different *Cannabis* extracts: CAN5, a Δ^9^-THC-rich extract; CAN9, a CBD-rich extract; and CAN10, a CBG-rich extract. Treatment with each of these *Cannabis* extracts for 24 h led to apoptosis of A549 cells in a dose-dependent manner (Figure 4A-4B). In order to further verify the proapoptotic effects of these extracts, we assessed caspase-3 cleavage of A549 cells by western blot analysis. Our findings showed induction of cleaved caspase-3 occurred following incubation of A549 cells for 24 h with either CAN5, CAN9, or CAN10 extracts (Figure 4C).

**Figure 5:**
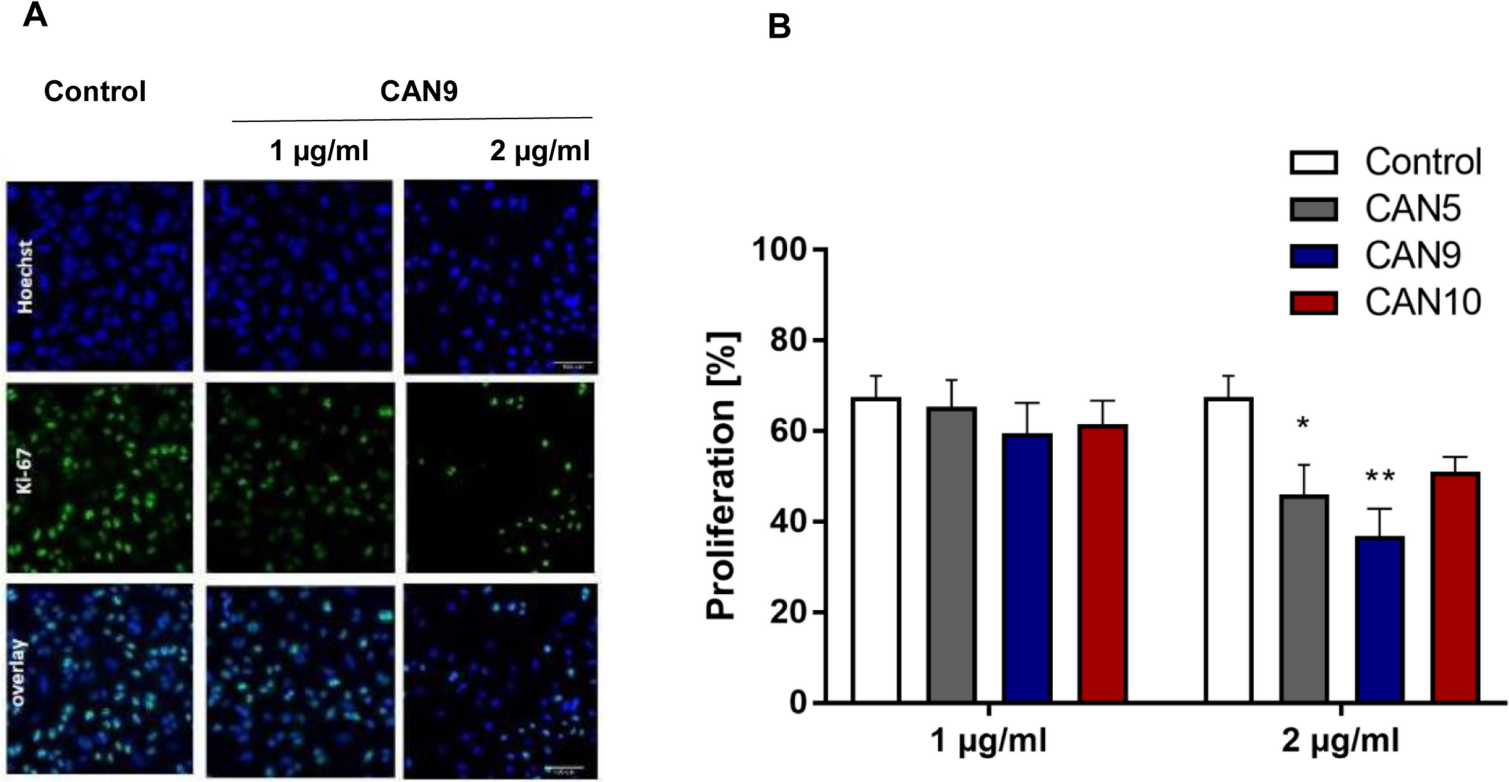
Anti-proliferative effect of *Cannabis* extracts on cancer cells. A549 cells were treated with 1-2 μg/ml CAN5, CAN9 or CAN10 extracts or control (DMSO) for 48 h. Cells were stained with anti-proliferation marker Ki67 antibody and counterstained with Hoechst. Percentage proliferation was calculated as % of Ki67-positive cells out of total cells. **(A)** Representative images of control and CAN9 treatment (1-2 μg/ml). Blue - Hoechst, green - Ki67, Turquoise - overlay of Hoechst and Ki67. **(B)** Bar chart presents % proliferation following incubation with CAN5, CAN9 or CAN10. Data are presented as mean ± SE (N=5). Asterisks indicate statistically significant differences between extract treatments vs. control. (*P < 0.05, **P < 0.005, ***P < 0.0005; two-way ANOVA with Bonferroni's post hoc multiple comparisons test).

We went on to test if *Cannabis* extracts not only promote cancer cell death rates but also inhibit cell proliferation*.* In order to assess the anti-proliferative effects of these extracts, we applied CAN5, CAN9 and CAN10 extracts onto A549 cells at concentrations (1-2 µg/ml) which we tested previously and found them to not affect cell survival (Figure 2). We found that after 48 h, 2 µg/ml of CAN5, CAN9, and CAN10 extracts reduced the percentage of A549 proliferating cells to 46.0, 36.7, and 51.0%, respectively, compared to 67.5% in the control (Figure 5A-5B). However, while the CAN5 and CAN9 extracts produced statistically significant reductions, the decrease following CAN10 application onto A549 cells was not statistically significant.

### Selectivity of *Cannabis* extracts on the survival of cancerous and non-cancerous human lung epithelial cells

**Figure 6:**
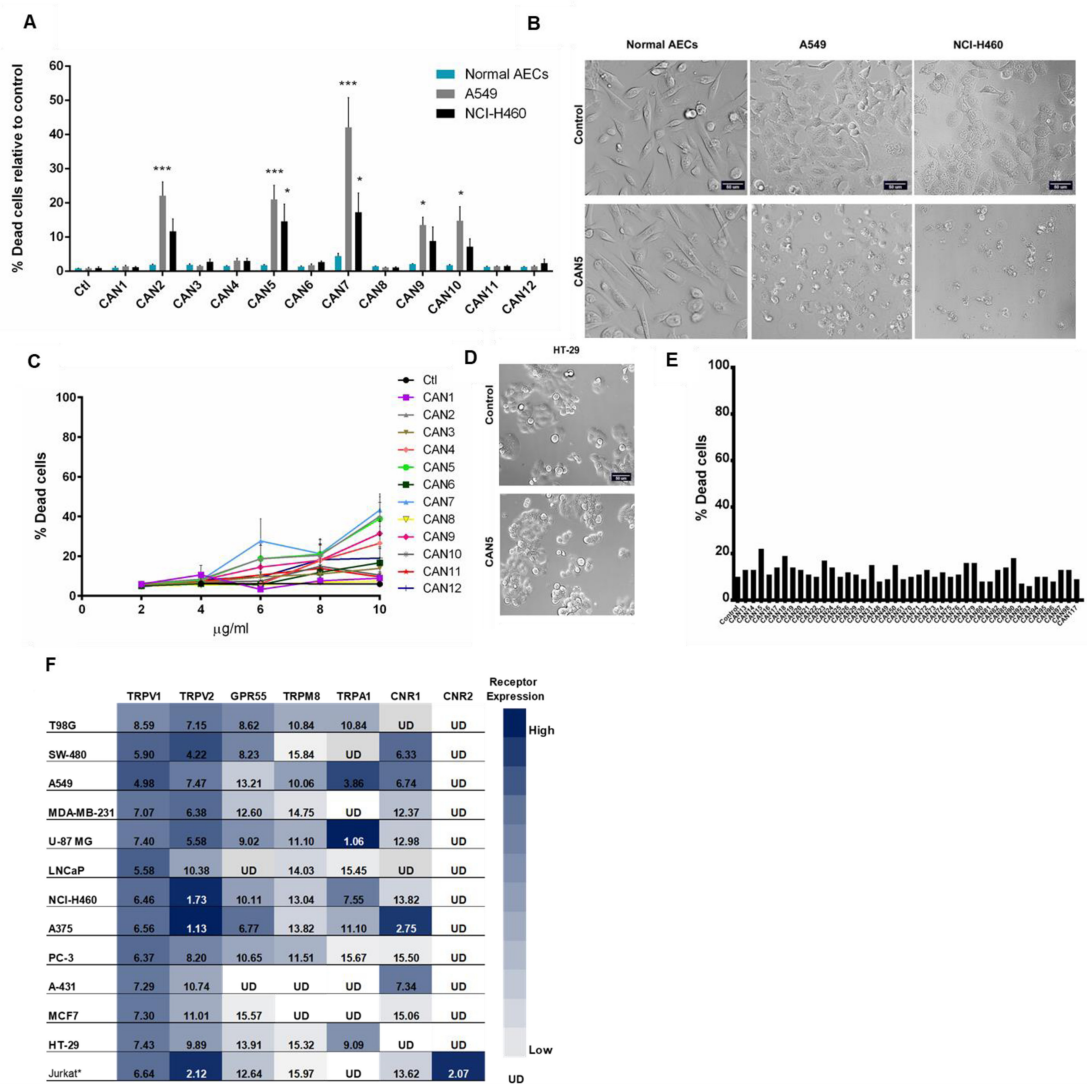
Selectivity of *Cannabis* extracts on the survival of human cells. **(A)** A549 and NCI-H460 lung carcinoma cells and normal bronchial epithelial cells (Normal AECs) were incubated with 4 µg/ml of CAN1-12 or control (ctl). Data are reported as mean ± SE of percentage of dead cells out of total cells relative to control treatment (N=3). Asterisks indicate statistical differences between cancer vs. normal AECs treated with *Cannabis* extracts (*P < 0.05, **P < 0.005, ***P < 0.0005; two-way ANOVA with Bonferroni's post hoc multiple comparisons test). **(B, D)** Representative brightfield images of normal AECs and lung carcinoma (NCI-H460, A549) (**B**) or HT-29 colon carcinoma (**D**) cell lines treated with control or CAN5. (**C**) Dose-response curve of HT-29 cells after 24 hours incubation with or without (control) 2-10 μg/ml of CAN1-CAN12. Data are presented as mean ± SE (N=9). **(E)** Screening results of 4 μg/ml of 43 different *Cannabis* extracts, or control on the survival of HT-29 cells (N=1). **(F)** Cannabimimetic receptors CNR1 (CB1), CNR2 (CB2), GPR55, TRPV1, TRPA1, TRPV2, TRPM8 mRNA levels were evaluated by qPCR. Expression levels were represented as ΔCT levels of the receptors. *Jurkat cell line was added as a control for CNR2 expression. Results are presented as mean expression of N=3 and normalized to the *GUSB* housekeeping gene. Values were color-coded according to the magnitude of expression. UD - Under Detectable Level. The higher the ΔCT values are the lower receptor expression.

Some research has shown that certain *Cannabis* preparations selectively promote cancer cell death better than non-cancerous cells [20, 31, 32]. Therefore, we tested this theory by applying *Cannabis* extracts CAN1-12 onto normal airway epithelial cells (AECs) and onto lung carcinoma A549 and NCI-H460 cancer cell lines. We found that the A549 and NCI-H460 cancer cell lines were statistically more sensitive to specific *Cannabis* extracts CAN2, CAN5, CAN7, CAN9, and CAN10 compared to normal AECs (Figure 6A-6B). CAN7, a Δ^9^-THC-rich extract, was the least discriminatory of the twelve extracts, as it significantly reduced the survival of both cancerous and non-cancer lung epithelial cell lines (Supplementary Figure 2).

As presented in this work, various cancer cell lines respond differently to the same *Cannabis* extracts. Of all the cell lines examined in this study, the HT-29 cell line was the least sensitive to all *Cannabis* extracts and concentrations tested (Figure 3A, 6C-6D). Remarkably, all extracts tested displayed weak potency against HT29 cells, as shown by IC50 values higher than 10 µg/mL (Figure 6C, Supplementary Table 1).

We therefore screened an additional 43 different extracts on HT-29 cell survival and none of them affected its cell survival at 4 µg/mL (Figure 6E). Thus, the HT-29 cell line possesses a unique characteristic or set of properties which relays relative resistance to *Cannabis* extract effects. One such property of the cells might be that they express a differing set of cannabimimetic receptors. As the expression of certain receptors in a specific cell line might affect its response to certain *Cannabis* extracts, we analyzed the mRNA levels of seven cannabimimetic receptors in human cancer cell lines using real-time qPCR. The mRNA of CNR1 (CB1), GPR55, TRPV1, TRPV2, TRPA1 and TRPM8 were differentially expressed in various cell lines grown *in vitro* (Figure 6F). TRPV1 and TRPV2 were expressed by all 12 tested cell lines (Figure 6F) while CNR1 was expressed by nine of the 12 cell lines examined. CNR1 levels in HT-29, LNCaP and T98G cells were not detected using the applied method.

CNR2 (CB2), was reported to be expressed mainly by immune cells, and therefore Jurkat T-cells were used as a positive control cell line for CNR2 expression. As expected CNR2 was found to be expressed only in the Jurkat cell line (Figure 6F). We further verified the CNR2 qPCR results by applying PCR onto the same cell lines and again found that only the Jurkat cell line expressed CNR2 (Supplementary Figure 3).

HT-29 and MCF7 cell lines were found to be the least sensitive to the 12 *Cannabis* extracts in this study, (Figure 3A and Supplementary Table 1). Moreover, HT-29 cell line was resistance to 43 additional different extracts at 4 µg/mL (Figure 6E). This was correlated to the low or under detectable expression levels of CNR1, GPR55 and TRPM8 in these cell lines.

### The correlation between phytocannabinoid profile and effect on cancer cell survival

**Figure 7:**
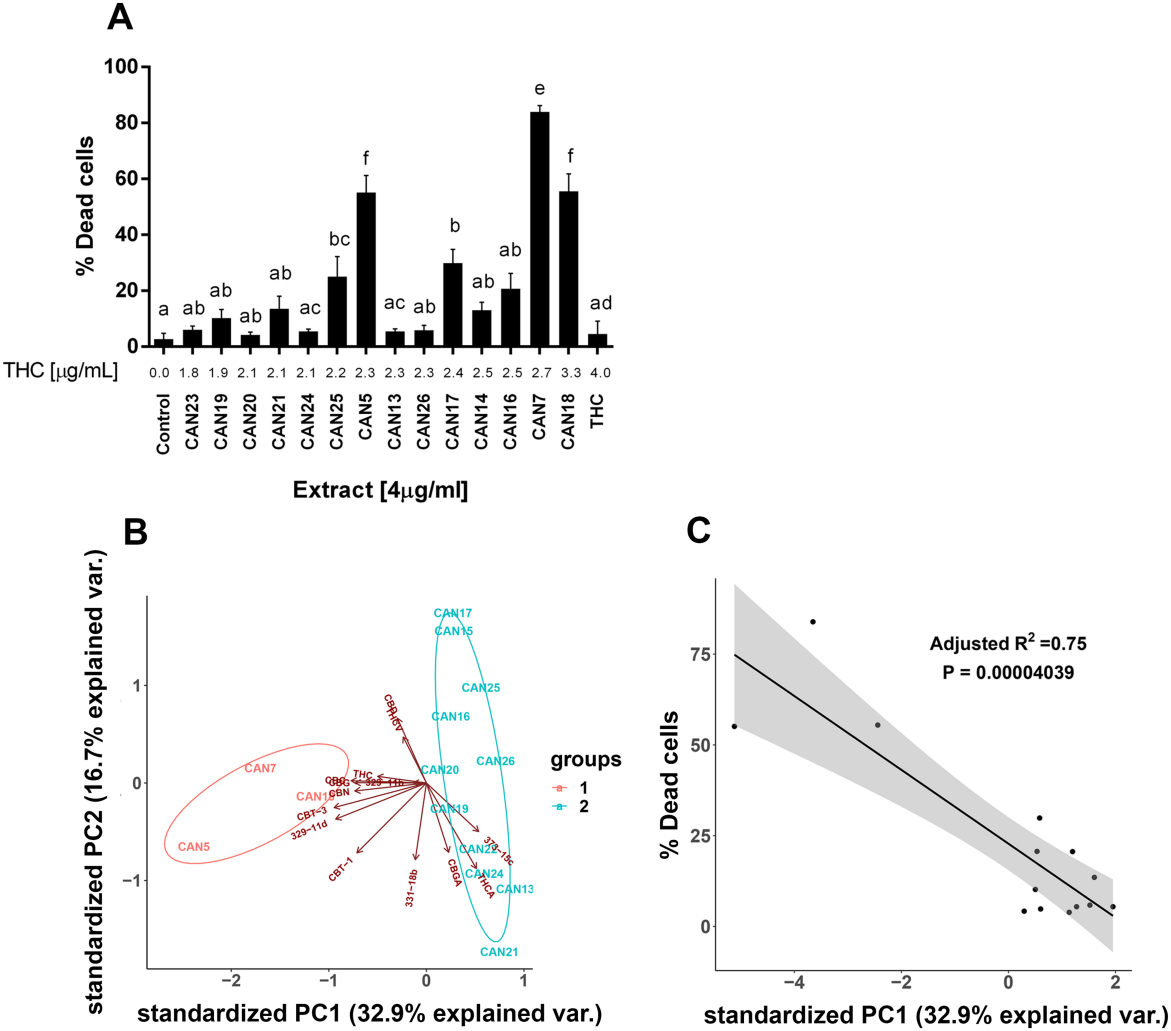
Differential effect of different high Δ9-THC extracts on the survival of cancer cells. **(A)** A549 cells were treated for 24 h with 4 µg/ml of 14 Δ^9^-THC-rich *Cannabis* extracts or pure Δ^9^-THC. Data are reported as mean ± SE of % dead cells out of total cells (N=5). Extracts are ordered on the x-axis by increasing Δ^9^-THC content in each extract (µg/ml). Statistical analysis was performed by one-way ANOVA, followed by Bonferroni's multiple comparisons test. Bars labelled with different letters are significantly different (*P* ≤ 0.05) from one another, according to the post-hoc ANOVA statistical analysis. **(B)** Principal Component Analysis (PCA) of 14 Δ^9^-THC-rich extracts according to the phytocannabinoid content. Criteria for cannabinoid inclusion in the PCA were detection in at least three extracts and a minimum concentration of 0.5 % w/w in one of the included extracts. **(C)** Linear regression of the percentage of dead A549 cells in response to 14 Δ^9^-THC-rich extracts according to their PC1 score.

We further examined which components of the *Cannabis* extracts determine their potency as an antitumor agent. CAN5 and CAN7 were found to be two of the most potent extracts by affecting the survival of most cell line tested and both contained high amounts of Δ^9^-THC (56.6 and 67.8%, respectively). Δ^9^-THC is one of the most common and well-studied components of the *Cannabis* plant and we suspected it might be the key factor in the antitumor effect of these two Δ^9^-THC-rich extracts. In order to answer this question, we examined the effects of pure Δ^9^-THC (>99%) along with 12 additional Δ^9^-THC-rich extracts (≥45% w/w Δ^9^-THC) from cluster 2 (Figure 1) on the A549 cell line (Figure 7A).

Although the 14 Δ^9^-THC-rich extracts seemed to possess similar phytocannabinoid profiles (Figure 1 and Supplementary Figure 4), they nonetheless exerted significantly different effects on the survival of the A549 cell line (Figure 7A and Supplementary Figure 5). For example, 4 µg/ml of both CAN5 and CAN13 extracts contained 2.3 µg/ml Δ^9^-THC, and yet these extracts induced an average of 55% and 5.5% of A549 dead cells, respectively. The effects of the chosen Δ^9^-THC-rich extracts on A549 cell survival was weakly correlated to the amount of Δ^9^-THC in the extracts (r=0.4553, *p*=0.0049). The effect of 4 µg/ml of purified Δ^9^-THC on A549 cell survival was not statistically different than the control. In fact, purified Δ^9^-THC was less potent in producing cell death than the Δ^9^-THC-rich extracts CAN5 and CAN7 on all the studied cancer cells lines (Supplementary Table 1). We then calculated the correlation between the percentages of dead cells to the amount of 13 other phytocannabinoids which were identified in these extracts (Supplementary Table 2). Each of these additional 13 phytocannabinoids showed a weak to moderate correlation to the percentage of dead A549 cells following incubation with Δ^9^-THC-rich extracts (Supplementary Table 2).

Next, we employed principal component analysis (PCA) to analyze the effects of the 14 Δ^9^-THC-rich extracts on A549 cell survival. This was done in order to identify which of the specific components were the most influential to the variance among the extracts, and to predict the extract's potency. For the PCA statistical analysis, each analyzed compound represented a variable while its quantity (% w/w) represented the observation. Figure 7B shows a bi-plot of the score and loading scatter plots of the first two principal components (PC1 and PC2). PC1 and PC2 were linear transformations of 14 identified phytocannabinoids and accounted for 47% of the total variance. According to the PC1 and PC2 scores in Figure 7B, the 14 Δ^9^-THC-rich extracts are divided into two main clusters, groups 1 and 2. Interestingly, group 1 clustered the three most potent extracts affecting A549 cell survival out of the 14 Δ^9^-THC-rich extracts: CAN5, CAN7 and CAN18 as summarized in Figure 7A. Furthermore, the effects of these Δ^9^-THC-rich extracts on A549 cell survival (described as percentage of dead cells) were highly correlated to the extracts' PC1 score (Figure 7C). This correlation to PC1 score was stronger than the correlation of the amount of each of the 14 phytocannabinoids alone in each extract to the effect of these extracts on A549 cell survival (Supplementary Table 2).

## DISCUSSION

*Cannabis* compounds have shown to exert anti-proliferative and proapoptotic effects on cancer cell lines as well as produce antitumor effects in experimental models of cancer [18–25]. Although there is great chemical diversity in *Cannabis* (Figure 1), most studies thus far, have focused on the therapeutic effects of only two prominent phytocannabinoids, Δ^9^-THC and CBD. This study demonstrates the anti-cancer activity of various whole *Cannabis* extracts on a set of human cancer cell lines. Our work clearly shows that whole-*Cannabis* extracts exhibit different and selective anti-cancer effects according to the varying toxicities observed for specific cell lines (Figures 3 and 6).

We found that specific *Cannabis* extracts such as CAN5, CAN9 and CAN10, which vary greatly in their phytocannabinoid profiles, significantly impaired the survival of various cancer cell lines tested (Figure 3 and Figure 4). Nevertheless, the studied cancer cell lines also differed in their susceptibilities towards the antitumor effects of various *Cannabis* extracts (Figure 3). Colon carcinoma HT-29 and breast carcinoma MCF-7 cell lines were the least sensitive to the *Cannabis* extracts used in this study (Figure 3 and Supplementary Table 1). The LNCaP prostate carcinoma cell line was exceptionally sensitive to extracts containing natural phytocannabinoids in their carboxylated form (e.g. Δ^9^-THCA, CBDA and others) (Figure 3). These results suggest that phytocannabinoids work through different pathways and receptors, which vary in different cancer cell populations.

As reviewed by *Pertwee RG et al*. (2010) [6], phytocannabinoids are known to produce certain physiological effects by binding to specific cannabimimetic receptors throughout the ECS. Previous reports indicated that Δ^9^-THC activates CB1 and GPR55 receptors [6, 33, 34]. However it was also recently shown that CB1 and GPR55 play opposing roles in colorectal cancer models in mice [15]. CBC and CBD were shown to be very potent activators of TRPA1 [10]. CBD was also reported as an antagonist or negative allosteric modulator of CB1 [35, 36]. Additional research has shown that Δ^9^-THC and CBD are able to activate TRPV2, TRPA1 and TRPM8 receptors [10, 37]. CBG was found to be a partial agonist of CB1, TRPA1, TRPV1 and TRPV2 receptors and an antagonist of TRPM8 [10, 38].

We therefore examined the mRNA expression of seven cannabimimetic receptors – CNR1 (CB1), CNR2 (CB2), GPR55, TRPV1, TRPV2, TRPA1, TRPM8 – and found them to be differentially expressed amongst the 12 tested cancer cell lines (Figure 6).

The presence or absence of these receptors in the tested cell lines may explain the differential potency of the extracts towards reducing cell survival. Both the HT-29 and MCF7 cell lines displayed low or under detectable expression levels of CNR1, GPR55 and TRPM8 (Figure 6F), which may explain why these cell lines were less responsive to *Cannabis* extracts.

It is important to mention that the lack of CB1 expression in HT29, LNcAP and T98G is in contradiction with previous works [39–41]. In addition, previous studies have shown that CB2 receptors are expressed by various cell lines including HT-29 [42], PC-3 [13] and LNCaP [41]. However, other studies [6, 43, 44] and observations found in the Human Protein Atlas [45] described that CB2 receptors are predominantly expressed by immune cells. In line with these studies, our qPCR and PCR results indicated CB2 mRNA expression to be under detectable levels in all examined cell lines, excluding the T lymphocyte Jurkat cells (Figure 6F and Supplementary Figure 3). These discrepancies might be a result of different methods used or a potential genomic evolution which might have occurred in these cell lines and resulted in gene expression variation [46].

Many previous reports highlight and demonstrate the anti-tumor effects of cannabinoids [23, 24, 32]. The majority of these reports were conducted with pure cannabinoids and thus ignored the potential effects of the whole preparation and its benefits. However, a few studies did suggest a synergistic effect between Δ^9^-THC and CBD. It has been reported that CBD neutralized Δ^9^-THC's adverse effects [47, 48] and that the combined administration of these two phytocannabinoids could act synergistically to reduce tumor growth [21, 28, 29]. Reinforcing this concept, *De Petrocellis et al.* (2011) demonstrated that some *Cannabis* extracts enriched with specific cannabinoids were more potent agonists of TRPA1, TRPV1, TRPV2 and TRPM8 receptors when compared to applying these same pure phytocannabinoids singularly [10]. These results support the supposition that beyond the major phytocannabinoids present in these extracts, other *Cannabis* extract components may play a role in either increasing phytocannabinoid potency or phytocannabinoid affinity to respective cannabimimetic receptors, and therefore are important for the anti-tumor effects produced by *Cannabis* [29, 49, 50].

Overall, we found that *Cannabis* extracts were very potent in producing cell death and some of these extracts were of Δ^9^-THC-rich type. However, in line with the studies mentioned above, we suggest that using whole *Cannabis* extracts is more effective in inducing cancer cell death than applying pure Δ^9^-THC on the studied cells lines. Furthermore, not all Δ^9^-THC-rich extracts produce the same effects when applied at the same concentrations on a specific cancer cell line. These findings indicate that compounds other than Δ^9^-THC in these extracts might act together in a polypharmacology way and determine the extract efficacy as antitumor agents (Figure 7, Supplementary Figure 5, Supplementary Tables 1 and 2).

Although we observed that specific Δ^9^-THC-rich *Cannabis* extracts were very potent in inducing cell death, their cytotoxic effects cannot be explained solely by the amount of Δ^9^-THC in the extracts. Nor can the potencies of these extracts be explained by other individual phytocannabinoids detected in them (Supplementary Table 2). Using PCA, we demonstrated that the combination of 14 specific phytocannabinoids identified in these extracts, may predict their potencies (Figure 7B). This PCA score scatter plot showed a clear division between the highly and less cytotoxic Δ^9^-THC-rich *Cannabis* extracts. The PC1 score was found to better describe the relationship between the extract composition and its cytotoxic effect on A549 cells compared to each phytocannabinoid alone (Supplementary Table 2 and Figure 7C). We suggest that this type of analysis might be used as a predictive tool for other Δ^9^-THC-rich *Cannabis* extracts in order to choose the most potent extract for a specific cancer cell type. The results from this research validate the need for comprehensive phytocannabinoid profiling and analysis for each *Cannabis* extract, as recently suggested in a publication from our group [30].

Taken as a whole, we concluded that medical *Cannabis* does not consist of a single therapeutic agent but rather a heterogeneous array of treatments. We propose that the fate of specific cancer cells following *Cannabis* extract application is dependent upon the synergistic effects of its phytocannabinoid composition, concentration applied, along with the cell specific characteristics (e.g. cannabimimetic receptor expression). Further research should investigate specific properties and mechanisms of cancer cell insensitivity to *Cannabis* extract effects. Future studies could focus on matching *Cannabis* extracts with specific phytocannabinoid compositions and their effects on specific cancer sub-types in order to optimize treatment effects. We hope that this study will lay the groundwork for future preclinical studies and randomized controlled clinical trials in order to provide evidence for effective *Cannabis* treatments for many cancer subtypes.

## MATERIALS AND METHODS

### Phytocannabinoid extraction and sample preparation

Air-dried medical *Cannabis* strains were obtained from several Israeli medical *Cannabis* distributors. *Cannabis* extracts were prepared as detailed in the Supplementary Materials. The extracts were reconstituted in DMSO to achieve a concentration of 50 mg/ml. For phytocannabinoid profiling, a fraction of the sample was diluted in DMSO to achieve final concentrations of 10, 1 and 0.1 µg/ml *Cannabis* extract to DMSO. Pure Δ^9^-THC (>99%) was acquired as a kind gift from Breath of Life (BOL) Pharma in Israel.

### Phytocannabinoid identification and quantification

Phytocannabinoid analyses were performed using a Thermo Scientific ultra-high-performance liquid chromatography (UHPLC) system coupled with a Q Exactive™ Focus Hybrid Quadrupole-Orbitrap MS (Thermo Scientific, Bremen, Germany). The chromatographic method and MS parameters are detailed in the Supplementary Materials. Identification and absolute quantification of phytocannabinoids was performed by external calibrations as described by Berman et al. (2018) [30].

### Cell cultures

Twelve well-characterized human adherent cancer cell lines from different solid tumor types were purchased from the American Type Culture Collection (ATCC, Manassas, VA, USA): MCF7 and MDA-MB-231 for breast adenocarcinoma; A375 for malignant melanoma; A-431 for epidermoid carcinoma; A549 and NCI-H460 for lung carcinoma; PC-3 for prostate adenocarcinoma; LNCaP for prostate carcinoma; SW480 and HT-29 for colorectal adenocarcinoma; U-87 MG and T98G for glioblastoma as well as primary normal bronchial/tracheal epithelial cells. Jurkat acute T cell leukemia cells were provided as a gift from Professor Yoram Reiter at the Technion, Israel Institute of Technology.

MCF-7, MDA-MB-231, A375, A-431, HT-29, U-87 MG and T98G cells were grown in high glucose DMEM (Sigma-Aldrich, D5796). A549, NCI-H460, PC-3, LNCaP, SW480 and Jurkat cells were grown in RPMI-1640 medium (Sigma-Aldrich, R8758) supplemented with 10% FBS (Biological Industries, 04-007-1A) and 100 units/ml of penicillin G and 100 μg/ml of streptomycin (Biological Industries, 03-031-1B). Primary normal cells were grown in serum-free conditions in Airway Epithelial Cell Basal Medium (ATCC, PCS 300-030) supplemented with the adequate cell growth kit (ATCC, PCS-300-040). All cells were maintained in a humidified atmosphere of 5% CO_2_ at 37 °C.

### Cell survival assay

Cells were cultured in 96-well plates, at 10,000 or 8,000 (for A549) cells/well in respective growth media. Following overnight incubation, growth media were replaced with media containing 0.5 % FBS. Different *Cannabis* extracts or pure Δ^9^-THC were added in triplicate at concentrations ranging from 2-20 µg/ml. DMSO was used as control and applied in the same amount as in the diluted extracts. Following 24 h incubation, the fluorescent probes propidium iodide (PI) (Sigma-Aldrich, P4864) and Hoechst (Thermo Fisher Scientific, H3570) were added to stain dead cells or all cells, respectively. Cells were visualized using an ImageXpress Micro® system (Molecular Devices, Sunnyvale, CA, USA). Four sites were imaged in each well and the number of detected signals per well was counted and analyzed by MetaXpress® software (Molecular Devices). Percentage of cell death was determined as the number of dead cells (stained with Hoechst and PI) divided by the total cells (stained with Hoechst), multiplied by 100.

### Cell apoptosis assay

Cells were cultured in six-well plates, at 3×10^5^ cells per well in respective growth media overnight. After overnight incubation, media were replaced with media containing 0.5 % FBS and *Cannabis* extracts or DMSO (control). All were then incubated for 24 h. Apoptotic cells were detected by annexin V/PI assay using flow cytometry or via detection of cleaved caspase-3 using a western blot assay as described below.

### Annexin V/PI assay

Apoptosis was assessed by annexin V-FITC (BioVision, 1006-200) and PI staining in annexin binding buffer (BioVision, 1006-100) according to the manufacturer’s instructions. 10,000 cells were acquired using a BD™ LSR II digital four-laser flow cytometer (BD Biosciences) and analyzed by BD FACSDiva™ software, version 6.1.2. (BD Biosciences). Results were calculated as the percentage of positive annexin V-FITC cells out of total cells counted.

### Cell lysis and western blot analyses

Following treatment, cells were solubilized in radioimmunoprecipitation assay buffer (Sigma-Aldrich, R0278) and protein concentration in lysates were determined using Bradford reagent (Sigma-Aldrich, B6916). Equal amounts of protein were resolved by Novex™ 4-20% Tris-Glycine Mini Gels (Thermo Fisher Scientific, XP04200BOX) and electrophoretically transferred to a nitrocellulose membrane (Bio-Rad, 1704159S). Membranes were blocked with Tris buffer saline (TBS) 0.1% Tween 20 buffer containing 5% BSA (Sigma-Aldrich, A7906) for one h. The blots were then incubated overnight at 4 ºC with anti-cleaved caspase-3 antibody (Cell Signaling Technology, 9664S) and β-tubulin (Cell Signaling Technology, clone D3U1W, 86298). This was followed by incubation with horseradish peroxidase (HRP) -labeled matching secondary antibodies. Immunoreactive bands were detected by Luminata™ HRP substrate (Millipore, WBLUR0500) and visualized using a MicroChemi imager (DNR Bioimaging Systems, Jerusalem, Israel).

### Immunofluorescence for Ki67 marker

A549 Cells were cultured in 96-well plates, at 8,000 cells/well in respective growth media. Following overnight incubation media were replaced with media containing 0.5% FBS and *Cannabis* extracts were added in sub-lethal concentrations, as determined in preliminary experiments (1-2 µg/ml). Extract were then incubated for 48 h in 37 °C with 5% CO_2_. Following this, cells were fixed using 4% paraformaldehyde, permeabilized using 0.1% Triton and blocked using 5% Normal Donkey Serum (Jackson ImmunoResearch Inc., 017-000-121) in PBS. Following blocking, cells were incubated with mouse anti-human Ki-67 antibody (BD Biosciences, 610969) overnight at 4°C. This was followed by incubation with Alexa Fluor® 488-conjugated donkey anti-mouse antibody (Thermo Fisher Scientific, A-21202) and counterstained with Hoechst (2 µg/ml). Cells were visualized with ImageXpress® Micro System at 10× magnification. The percentage of proliferation was calculated as the number of Ki-67 positive cells divided by the number of total cells counted in each well, multiplied by 100.

### RNA extraction

Total RNA was isolated from cells (1×10^6^ cells/sample) using Trizol® (Thermo Fisher Scientific, 15596026) and RNeasy kit (Qiagen, 74104) according to the manufacturers’ instructions. Sample quality was assessed by both spectrophotometer (Nanodrop Technologies®, Wilmington, DE, USA) and agarose gels (1%).

### Real-time quantitative PCR

cDNA was synthesized from 1 µg of RNA with the qScript™ cDNA synthesis kit (Quanta Biosciences, 95047) according to the manufacturer’s instructions. The mRNA expression levels of human receptors CNR1 (CB1), CNR2 (CB2), GPR55, TRPV1, TRPV2, TRPM8 and TRPA1 were quantified using TaqMan® Gene Expression assays (Applied Biosystems - Thermo Fisher Scientific, 4448892) and a quantitative-PCR 7300 system (Applied Biosystems - Thermo Fisher Scientific). Relative expression values were normalized using an endogenous housekeeping gene GUSB control and calculated using standard Δ-Ct methods.

### Statistical analysis

Resulting LC-MS data of cannabinoid content (% w/w) was subjected to unsupervised hierarchical clustering analysis using R software [51] and the pheatmap package [52]. PCA was created to provide a visual depiction of the variation in phytocannabinoid compositions among extracts using R software and the ggbiplot package [53]. Linear regression was done using R software.

All other statistical analyses were conducted using GraphPad Prism software version 7.04 (GraphPad Inc.). Data were reported as the mean ± SEM of at least three independent experiments. Multiple groups were compared using one-way or two-way ANOVA followed by Bonferroni post-hoc multiple comparisons test. A value of at least *P* ≤ 0.05 was considered significant for all tests.

## SUPPLEMENTARY MATERIALS FIGURE AND TABLES



## Abbreviations

Δ^9^-THC: Δ^9^-*trans*-tetrahydrocannabinol
CBD: cannabidiol
ECS: endocannabinoid system
CB1: cannabinoid receptor type 1
CB2: cannabinoid receptor type 2
GPR55: G protein-coupled receptor 55
TRP: transient receptor potential
TRPV1: TRPV2: TRP cation channel subfamily V member 1,2
TRPA1: TRP cation channel subfamily A, member 1
TRPM8: TRP cation channel subfamily M member 8
UHPLC: ultra-high-performance liquid chromatography
PI: propidium iodide
TBS: Tris buffer saline
HRP: horseradish peroxidase
ESI-LC/MS: electrospray ionization liquid chromatography mass spectrometry
CBDA: cannabidiolic acid
CBEA: cannabielsoic acid
CBC: cannabichromene
Δ^9^-THCA: Δ^9^-*trans*-tetrahydrocannabinolic acid
CBNA: cannabinolic acid
CBTA: (±)-*trans/cis*-cannabitriolic acid
CBG: cannabigerol
AECs: airway epithelial cells
RPMI: Roswell Park Memorial Institute
PCA: Principal Component Analysis

## References

[1] Hanuš LO, Meyer SM, Muñoz E, Taglialatela-Scafati O, Appendino G. Phytocannabinoids: a unified critical inventory. Nat Prod Rep. 2016; 33:1357–92. 10.1039/C6NP00074F. . 27722705

[2] Mechoulam R, Gaoni Y. Hashish. IV. The isolation and structure of cannabinolic cannabidiolic and cannabigerolic acids. Tetrahedron. 1965; 21:1223–29. 10.1016/0040-4020(65)80064-3. . 5879350

[3] D'Souza DC, Perry E, MacDougall L, Ammerman Y, Cooper T, Wu YT, Braley G, Gueorguieva R, Krystal JH. The psychotomimetic effects of intravenous delta-9- tetrahydrocannabinol in healthy individuals: implications for psychosis. Neuropsychopharmacology. 2004; 29:1558–72. 10.1038/sj.npp.1300496. . 15173844

[4] Bergamaschi MM, Queiroz RH, Zuardi AW, Crippa JA. Safety and side effects of cannabidiol, a Cannabis sativa constituent. Curr Drug Saf. 2011; 6:237–49. 10.2174/157488611798280924. . 22129319

[5] Izzo AA, Borrelli F, Capasso R, Di Marzo V, Mechoulam R. Non-psychotropic plant cannabinoids: new therapeutic opportunities from an ancient herb. Trends Pharmacol Sci. 2009; 30:515–27. 10.1016/j.tips.2009.07.006. . 19729208

[6] Pertwee RG, Howlett AC, Abood ME, Alexander SP, Di Marzo V, Elphick MR, Greasley PJ, Hansen HS, Kunos G, Mackie K, Mechoulam R, Ross RA. International Union of Basic and Clinical Pharmacology. LXXIX. Cannabinoid receptors and their ligands: beyond CB_1_ and CB_2_. Pharmacol Rev. 2010; 62:588–631. 10.1124/pr.110.003004. . 21079038 PMC2993256

[7] Pacher P, Bátkai S, Kunos G. The endocannabinoid system as an emerging target of pharmacotherapy. Pharmacol Rev. 2006; 58:389–462. 10.1124/pr.58.3.2. . 16968947 PMC2241751

[8] McPartland JM. Phylogenomic and chemotaxonomic analysis of the endocannabinoid system. Brain Res Brain Res Rev. 2004; 45:18–29. 10.1016/j.brainresrev.2003.11.005. . 15063097

[9] Pertwee RG. GPR55: a new member of the cannabinoid receptor clan? Br J Pharmacol. 2007; 152:984–86. 10.1038/sj.bjp.0707464. . 17876300 PMC2095104

[10] De Petrocellis L, Ligresti A, Moriello AS, Allarà M, Bisogno T, Petrosino S, Stott CG, Di Marzo V. Effects of cannabinoids and cannabinoid-enriched Cannabis extracts on TRP channels and endocannabinoid metabolic enzymes. Br J Pharmacol. 2011; 163:1479–94. 10.1111/j.1476-5381.2010.01166.x. . 21175579 PMC3165957

[11] Leishman E, Bradshaw HB. Chapter 3 - N-Acyl Amides: Ubiquitous Endogenous Cannabimimetic Lipids That Are in the Right Place at the Right Time. The Endocannabinoidome. 2015 pp. 33–48. 10.1016/B978-0-12-420126-2.00003-1.

[12] Wang D, Wang H, Ning W, Backlund MG, Dey SK, DuBois RN. Loss of cannabinoid receptor 1 accelerates intestinal tumor growth. Cancer Res. 2008; 68:6468–76. 10.1158/0008-5472.CAN-08-0896. . 18676872 PMC2561258

[13] Orellana-Serradell O, Poblete CE, Sanchez C, Castellón EA, Gallegos I, Huidobro C, Llanos MN, Contreras HR. Proapoptotic effect of endocannabinoids in prostate cancer cells. Oncol Rep. 2015; 33:1599–608. 10.3892/or.2015.3746. . 25606819 PMC4358087

[14] Pérez-Gómez E, Andradas C, Blasco-Benito S, Caffarel MM, García-Taboada E, Villa-Morales M, Moreno E, Hamann S, Martín-Villar E, Flores JM, Wenners A, Alkatout I, Klapper W, et al. Role of cannabinoid receptor CB2 in HER2 pro-oncogenic signaling in breast cancer. J Natl Cancer Inst. 2015; 107:djv077. 10.1093/jnci/djv077. . 25855725

[15] Hasenoehrl C, Feuersinger D, Sturm EM, Bärnthaler T, Heitzer E, Graf R, Grill M, Pichler M, Beck S, Butcher L, Thomas D, Ferreirós N, Schuligoi R, et al. G protein-coupled receptor GPR55 promotes colorectal cancer and has opposing effects to cannabinoid receptor 1. Int J Cancer. 2018; 142:121–32. 10.1002/ijc.31030. . 28875496 PMC5679368

[16] Larrinaga G, Sanz B, Pérez I, Blanco L, Cándenas ML, Pinto FM, Gil J, López JI. Cannabinoid CB1receptor is downregulated in clear cell renal cell carcinoma. J Histochem Cytochem. 2010; 58:1129–34. 10.1369/jhc.2010.957126. . 20852034 PMC2989249

[17] Maccarrone M, Lorenzon T, Bari M, Melino G, Finazzi-Agro A. Anandamide induces apoptosis in human cells via vanilloid receptors. Evidence for a protective role of cannabinoid receptors. J Biol Chem. 2000; 275:31938–45. 10.1074/jbc.M005722200. . 10913156

[18] Galanti G, Fisher T, Kventsel I, Shoham J, Gallily R, Mechoulam R, Lavie G, Amariglio N, Rechavi G, Toren A. Delta 9-tetrahydrocannabinol inhibits cell cycle progression by downregulation of E2F1 in human glioblastoma multiforme cells. Acta Oncol. 2008; 47:1062–70. 10.1080/02841860701678787. . 17934890

[19] Solinas M, Massi P, Cinquina V, Valenti M, Bolognini D, Gariboldi M, Monti E, Rubino T, Parolaro D. Cannabidiol, a non-psychoactive cannabinoid compound, inhibits proliferation and invasion in U87-MG and T98G glioma cells through a multitarget effect. PLoS One. 2013; 8:e76918. 10.1371/journal.pone.0076918. . 24204703 PMC3804588

[20] Galve-Roperh I, Sánchez C, Cortés ML, Gómez del Pulgar T, Izquierdo M, Guzmán M. Anti-tumoral action of cannabinoids: involvement of sustained ceramide accumulation and extracellular signal-regulated kinase activation. Nat Med. 2000; 6:313–19. 10.1038/73171. . 10700234

[21] Armstrong JL, Hill DS, McKee CS, Hernandez-Tiedra S, Lorente M, Lopez-Valero I, Eleni Anagnostou M, Babatunde F, Corazzari M, Redfern CP, Velasco G, Lovat PE. Exploiting cannabinoid-induced cytotoxic autophagy to drive melanoma cell death. J Invest Dermatol. 2015; 135:1629–37. 10.1038/jid.2015.45. . 25674907

[22] McAllister SD, Murase R, Christian RT, Lau D, Zielinski AJ, Allison J, Almanza C, Pakdel A, Lee J, Limbad C, Liu Y, Debs RJ, Moore DH, Desprez PY. Pathways mediating the effects of cannabidiol on the reduction of breast cancer cell proliferation, invasion, and metastasis. Breast Cancer Res Treat. 2011; 129:37–47. 10.1007/s10549-010-1177-4. . 20859676 PMC3410650

[23] Ligresti A, Moriello AS, Starowicz K, Matias I, Pisanti S, De Petrocellis L, Laezza C, Portella G, Bifulco M, Di Marzo V. Antitumor activity of plant cannabinoids with emphasis on the effect of cannabidiol on human breast carcinoma. J Pharmacol Exp Ther. 2006; 318:1375–87. 10.1124/jpet.106.105247. . 16728591

[24] Velasco G, Sánchez C, Guzmán M. Towards the use of cannabinoids as antitumour agents. Nat Rev Cancer. 2012; 12:436–44. 10.1038/nrc3247. . 22555283

[25] Borrelli F, Pagano E, Romano B, Panzera S, Maiello F, Coppola D, De Petrocellis L, Buono L, Orlando P, Izzo AA. Colon carcinogenesis is inhibited by the TRPM8 antagonist cannabigerol, a Cannabis-derived non-psychotropic cannabinoid. Carcinogenesis. 2014; 35:2787–97. 10.1093/carcin/bgu205. . 25269802

[26] Pertwee RG. Emerging strategies for exploiting cannabinoid receptor agonists as medicines. Br J Pharmacol. 2009; 156:397–411. 10.1111/j.1476-5381.2008.00048.x. . 19226257 PMC2697681

[27] Williamson EM. Synergy and other interactions in phytomedicines. Phytomedicine. 2001; 8:401–09. 10.1078/0944-7113-00060. . 11695885

[28] Nabissi M, Morelli MB, Offidani M, Amantini C, Gentili S, Soriani A, Cardinali C, Leoni P, Santoni G. Cannabinoids synergize with carfilzomib, reducing multiple myeloma cells viability and migration. Oncotarget. 2016; 7:77543–57. 10.18632/oncotarget.12721. . 27769052 PMC5363603

[29] Blasco-Benito S, Seijo-Vila M, Caro-Villalobos M, Tundidor I, Andradas C, García-Taboada E, Wade J, Smith S, Guzmán M, Pérez-Gómez E, Gordon M, Sánchez C. Appraising the "entourage effect": antitumor action of a pure cannabinoid versus a botanical drug preparation in preclinical models of breast cancer. Biochem Pharmacol. 2018; 157:285–93. 10.1016/j.bcp.2018.06.025. . 29940172

[30] Berman P, Futoran K, Lewitus GM, Mukha D, Benami M, Shlomi T, Meiri D. A new ESI-LC/MS approach for comprehensive metabolic profiling of phytocannabinoids in Cannabis. Sci Rep. 2018; 8:14280. 10.1038/s41598-018-32651-4. . 30250104 PMC6155167

[31] Blázquez C, Carracedo A, Barrado L, Real PJ, Fernández-Luna JL, Velasco G, Malumbres M, Guzmán M. Cannabinoid receptors as novel targets for the treatment of melanoma. FASEB J. 2006; 20:2633–35. 10.1096/fj.06-6638fje. . 17065222

[32] Romano B, Borrelli F, Pagano E, Cascio MG, Pertwee RG, Izzo AA. Inhibition of colon carcinogenesis by a standardized Cannabis sativa extract with high content of cannabidiol. Phytomedicine. 2014; 21:631–39. 10.1016/j.phymed.2013.11.006. . 24373545

[33] Matsuda LA, Lolait SJ, Brownstein MJ, Young AC, Bonner TI. Structure of a cannabinoid receptor and functional expression of the cloned cDNA. Nature. 1990; 346:561–64. 10.1038/346561a0. . 2165569

[34] Ryberg E, Larsson N, Sjögren S, Hjorth S, Hermansson NO, Leonova J, Elebring T, Nilsson K, Drmota T, Greasley PJ. The orphan receptor GPR55 is a novel cannabinoid receptor. Br J Pharmacol. 2007; 152:1092–101. 10.1038/sj.bjp.0707460. . 17876302 PMC2095107

[35] Thomas A, Baillie GL, Phillips AM, Razdan RK, Ross RA, Pertwee RG. Cannabidiol displays unexpectedly high potency as an antagonist of CB1 and CB2 receptor agonists in vitro. Br J Pharmacol. 2007; 150:613–23. 10.1038/sj.bjp.0707133. . 17245363 PMC2189767

[36] Laprairie RB, Bagher AM, Kelly ME, Denovan-Wright EM. Cannabidiol is a negative allosteric modulator of the cannabinoid CB1 receptor. Br J Pharmacol. 2015; 172:4790–805. 10.1111/bph.13250. . 26218440 PMC4621983

[37] De Petrocellis L, Vellani V, Schiano-Moriello A, Marini P, Magherini PC, Orlando P, Di Marzo V. Plant-Derived cannabinoids modulate the activity of transient receptor potential channels of ankyrin type-1 and melastatin type-8. J Pharmacol Exp Ther. 2008; 325:1007–15. 10.1124/jpet.107.134809. . 18354058

[38] Cascio MG, Gauson LA, Stevenson LA, Ross RA, Pertwee RG. Evidence that the plant cannabinoid cannabigerol is a highly potent α2-adrenoceptor agonist and moderately potent 5HT1A receptor antagonist. Br J Pharmacol. 2010; 159:129–41. 10.1111/j.1476-5381.2009.00515.x. . 20002104 PMC2823359

[39] Cianchi F, Papucci L, Schiavone N, Lulli M, Magnelli L, Vinci MC, Messerini L, Manera C, Ronconi E, Romagnani P, Donnini M, Perigli G, Trallori G, et al. Cannabinoid receptor activation induces apoptosis through tumor necrosis factor alpha-mediated ceramide de novo synthesis in colon cancer cells. Clin Cancer Res. 2008; 14:7691–700. 10.1158/1078-0432.CCR-08-0799. . 19047095

[40] Lorente M, Torres S, Salazar M, Carracedo A, Hernández-Tiedra S, Rodríguez-Fornés F, García-Taboada E, Meléndez B, Mollejo M, Campos-Martín Y, Lakatosh SA, Barcia J, Guzmán M, Velasco G. Stimulation of the midkine/ALK axis renders glioma cells resistant to cannabinoid antitumoral action. Cell Death Differ. 2011; 18:959–73. 10.1038/cdd.2010.170. . 21233844 PMC3131933

[41] Sarfaraz S, Afaq F, Adhami VM, Mukhtar H. Cannabinoid receptor as a novel target for the treatment of prostate cancer. Cancer Res. 2005; 65:1635–41. 10.1158/0008-5472.CAN-04-3410. . 15753356

[42] Wright K, Rooney N, Feeney M, Tate J, Robertson D, Welham M, Ward S. Differential expression of cannabinoid receptors in the human colon: cannabinoids promote epithelial wound healing. Gastroenterology. 2005; 129:437–53. 10.1016/j.gastro.2005.05.026. . 16083701

[43] Galiègue S, Mary S, Marchand J, Dussossoy D, Carrière D, Carayon P, Bouaboula M, Shire D, Le Fur G, Casellas P. Expression of central and peripheral cannabinoid receptors in human immune tissues and leukocyte subpopulations. Eur J Biochem. 1995; 232:54–61. 10.1111/j.1432-1033.1995.tb20780.x. . 7556170

[44] Brown SM, Wager-Miller J, Mackie K. Cloning and molecular characterization of the rat CB2 cannabinoid receptor. Biochim Biophys Acta. 2002; 1576:255–64. 10.1016/S0167-4781(02)00341-X. 12084572

[45] Uhlén M, Fagerberg L, Hallström BM, Lindskog C, Oksvold P, Mardinoglu A, Sivertsson Å, Kampf C, Sjöstedt E, Asplund A, Olsson I, Edlund K, Lundberg E, et al. Proteomics. Tissue-based map of the human proteome. Science. 2015; 347:1260419–1260419. 10.1126/science.1260419. . 25613900

[46] Ben-David U, Siranosian B, Ha G, Tang H, Oren Y, Hinohara K, Strathdee CA, Dempster J, Lyons NJ, Burns R, Nag A, Kugener G, Cimini B, et al. Genetic and transcriptional evolution alters cancer cell line drug response. Nature. 2018; 560:325–30. 10.1038/s41586-018-0409-3. . 30089904 PMC6522222

[47] Zuardi AW, Shirakawa I, Finkelfarb E, Karniol IG. Action of cannabidiol on the anxiety and other effects produced by delta 9-THC in normal subjects. Psychopharmacology (Berl). 1982; 76:245–50. 10.1007/BF00432554. . 6285406

[48] Russo E, Guy GW. A tale of two cannabinoids: the therapeutic rationale for combining tetrahydrocannabinol and cannabidiol. Med Hypotheses. 2006; 66:234–46. 10.1016/j.mehy.2005.08.026. . 16209908

[49] Russo EB. Taming THC: potential cannabis synergy and phytocannabinoid-terpenoid entourage effects. Br J Pharmacol. 2011; 163:1344–64. 10.1111/j.1476-5381.2011.01238.x. . 21749363 PMC3165946

[50] Wagner H, Ulrich-Merzenich G. Synergy research: approaching a new generation of phytopharmaceuticals. Phytomedicine. 2009; 16:97–110. 10.1016/j.phymed.2008.12.018. . 19211237

[51] R Core Team (2018) R: A language and environment for statistical computing. R Foundation for Statistical Computing, Vienna, Austria Available online at https://www.R-project.org/.

[52] Kolde R. (2018) pheatmap: Pretty Heatmaps. R package version 1.0.10. https://CRAN.R-project.org/package=pheatmap.

[53] Vu VQ. (2011) ggbiplot: A ggplot2 based biplot. R package version 0.55. https://github.com/vqv/ggbiplot.

