# Supplementary material for: The heterogeneity and complexity of Cannabis extracts as antitumor agents
Source: Oncotarget. 2019 Jun 25;10(41):4091–106. doi: 10.18632/oncotarget.26983 (PMC6609248; doi:10.18632/oncotarget.26983)
Supplement: Supplementary file 1 [file oncotarget-10-4091-s001.pdf]

The heterogeneity and complexity of *Cannabis* extracts as antitumor agents

SUPPLEMENTARY MATERIALS

PHYTOCANNABINOID EXTRACTION AND SAMPLE PREPARATION

Cannabis plant samples were ground to a fine powder using an electrical grinder. Prior to phytocannabinoid extraction, several of the flowers underwent heat-decarboxylation in an oven at 120 °C for 1 h. Approximately 5 g of the natural or decarboxylated flowers were accurately weighed and extracted with 50 ml ethanol. Samples were sonicated in an ultrasonic bath for 30 min and then agitated in an orbital shaker at 25 °C for 15 min. Samples were then filtered under pressure through Whatman filter paper number 4 and the ethanol was evaporated under reduced pressure at 38 °C using a rotary evaporator (Laborata 4000; Heidolph Instruments GmbH & Co. KG; Germany).

PHYTOCANNABINOID IDENTIFICATION AND QUANTIFICATION

Phytocannabinoid analyses were performed using a Thermo Scientific ultra-high-performance liquid chromatography (UHPLC) system coupled with a Q Exactive™ Focus Hybrid Quadrupole-Orbitrap MS (Thermo Scientific, Bremen, Germany). The chromatographic separation was achieved using a Halo C18 Fused Core column (2.7 µm, 150 mm × 2.1 mm i.d.) with a guard column (2.7 µm, 5 mm × 2.1 mm i.d) (Advanced Materials Technology, Delaware, USA) and a ternary A/B/C multistep gradient (solvent A: 0.1 % acetic acid in Milli Q water, solvent B: 0.1% acetic acid in acetonitrile, and solvent C: methanol, all solvents were of LC/MS grade). The multistep gradient program was established as follows: initial conditions were 50% B raised to 67% B until 3 min, held at 67% B for 5 min, and then raised to 90% B until 12 min, held at 90 % B until 15 min, decreased to 50% B over the next min, and held at 50 % B until 20 min for re-equilibration of the system prior to the next injection. Solvent C was initially 5% and then lowered to 3% until 3 min, held at 3 % until 8 min, raised to 5 % until 12 min and then kept constant at 5 % throughout the run. A flow rate of 0.25 ml/min was used, the column temperature was 30 °C and the injection volume was 1 µL. MS acquisition was carried out with a heated electro spray ionization (HESI-II) ion source operated in negative mode. Source parameters were as follows: sheath gas flow rate, auxiliary gas flow rate and sweep gas flow rate: 50, 20 and 0 arbitrary units respectively; capillary temperature: 350 °C; heater temperature: 50 °C; spray voltage: 3.00 kV. The scan range was 150–550 m/z for all acquisition events. MS was operated in full MS^1 mode at 70,000 resolution, and the AGC target was set to 10^6 with a maximum IT of 100 ms.

POLYMERASE CHAIN REACTION (PCR) ANALYSIS

mRNA expression levels of the human CNR2 gene (CB2) were quantified in 50 ng cDNA using Red Load Taq Master PCR assay. PCR amplification was performed using the following primers: Forward: atggaggaatgctgggtgacagaga Reverse: gcaatcagagaggtct agatctctggaatct. Results were visualized using 1.2% gel electrophoresis.

Type	Compound	CAN6	CAN4	CAN11	CAN7	CAN5	CAN12	CAN2	CAN9	CAN3	CAN8	CAN1	CAN10
Δ^9-THCtype	THCA	55.0065	48.0668	29.9010	0.0000	0.3049	0.2170	0.0000	0.0000	0.0000	1.1003	16.6407	0.0000
	THC	6.1633	15.9636	1.7548	67.8032	56.5847	35.1295	25.9923	2.3489	1.4977	0.0000	3.1207	0.0000
	THCA-C4	0.3514	0.2030	0.1169	0.0000	0.0000	0.0000	0.0000	0.0000	0.0000	0.0000	0.0665	0.0000
	THC-C4	0.0000	0.0000	0.0000	0.1969	0.1085	0.0000	0.0000	0.0000	0.0000	0.0000	0.0000	0.0000
	THCVA	1.1172	0.3818	0.3895	0.0000	0.0000	0.0000	0.0000	0.0000	0.0000	0.0360	0.1295	0.0000
	THCV	0.0000	0.0000	0.0000	0.6137	0.2072	0.2246	0.0000	0.0000	0.0000	0.0000	0.0000	0.0000
	THCOA	0.2371	0.3631	0.0676	0.0000	0.0000	0.0000	0.0000	0.0000	0.0000	0.0000	0.1178	0.0000
	THCMA	0.0617	0.0896	0.0297	0.0000	0.0000	0.0000	0.0000	0.0000	0.0000	0.0000	0.0351	0.0000
CBDtype	CBDA	0.1922	0.2139	0.0787	0.0000	0.0000	0.0093	0.1294	6.2145	0.0249	42.4938	31.1696	0.0848
	CBD	0.0000	0.0000	0.0000	0.2107	0.2125	0.0776	35.8411	55.5851	27.1263	4.4839	1.8886	0.8097
	CBDA-C4	0.0000	0.0000	0.0000	0.0000	0.0015	0.0000	0.0023	0.0206	0.0000	0.1345	0.0466	0.0000
	CBD-C4	0.0000	0.0000	0.0000	0.0000	0.0000	0.0000	0.0564	0.1803	0.0521	0.0172	0.0000	0.0000
	CBDVA	0.0014	0.0000	0.0000	0.0000	0.0000	0.0000	0.0000	0.0701	0.0000	0.4627	0.0433	0.0000
	CBDV	0.0000	0.0000	0.0000	0.0000	0.0000	0.0000	0.0589	0.7227	0.1896	0.0573	0.0000	0.0210
	CBDOA	0.0000	0.0000	0.0000	0.0000	0.0000	0.0000	0.0000	0.0085	0.0000	0.0307	0.0085	0.0000
CBGtype	CBGA	12.7114	4.4696	0.3930	0.2240	0.0725	0.0739	0.0000	0.1960	0.0000	0.8667	1.1989	0.0196
	CBG	0.8920	0.6684	0.1866	12.6179	4.4623	0.5928	1.8095	1.7364	1.9181	0.3457	0.1926	54.6694
	CBGA-C4	0.0192	0.0222	0.0026	0.0362	0.0408	0.0114	0.0131	0.0000	0.0000	0.0034	0.0032	0.0000
	CBG-C4	0.0000	0.0000	0.0000	0.0094	0.0000	0.0000	0.0000	0.0000	0.0000	0.0000	0.0000	0.1466
	CBGV	0.0000	0.0000	0.0000	0.0071	0.0000	0.0000	0.0000	0.0000	0.0000	0.0000	0.0000	0.8819
	CBGO	0.0000	0.0000	0.0000	0.0000	0.0000	0.0000	0.0000	0.0000	0.0000	0.0000	0.0000	0.0651
	Sesqui-CBG	0.0130	0.0277	0.0110	0.0160	0.0310	0.0187	0.0273	0.0374	0.0350	0.0173	0.0161	0.0358
CBCtype	CBCA	0.3390	0.4440	0.1316	0.0061	0.0060	0.0252	0.0031	0.0763	0.0000	0.6815	0.6925	0.0000
	CBC	0.0749	0.2113	0.0163	0.8269	1.0927	0.3350	1.9335	2.4134	1.5208	0.1785	0.1491	0.7137
	CBCVA	0.0042	0.0000	0.0000	0.0000	0.0000	0.0000	0.0000	0.0031	0.0000	0.0277	0.0037	0.0000
	CBCV	0.0000	0.0000	0.0000	0.0000	0.0000	0.0000	0.0000	0.0880	0.0255	0.0000	0.0000	0.0417
CBN type	CBNA	0.7241	1.3076	0.4984	0.0000	0.0121	0.0000	0.0000	0.0000	0.0000	0.0488	0.2313	0.0000
	CBN	0.0417	0.3022	0.0171	1.0339	2.7877	0.3699	1.0495	0.0830	1.5111	0.0143	0.0252	0.0000
CBE type	CBEA	0.0109	0.0105	0.0000	0.0000	0.0000	0.0000	0.0000	0.0362	0.0000	0.1430	0.0837	0.0000
	CBE	0.0000	0.0000	0.0000	0.0000	0.0000	0.0000	0.2917	0.2958	0.9250	0.0156	0.0000	0.0543
	CBNDA	0.0000	0.0000	0.0000	0.0000	0.0000	0.0000	0.0000	0.0043	0.0000	0.0651	0.0296	0.0000
	CBL	0.0000	0.0000	0.0000	0.0000	0.0458	0.0000	0.0582	0.0386	0.0848	0.0000	0.0000	0.0444
CBTtype	CBTA-1	1.5635	1.2756	1.8236	0.0000	0.0166	0.0037	0.0000	0.0000	0.0000	0.0466	0.3494	0.0000
	CBT-1	1.0314	2.6370	0.6330	3.3258	4.1060	1.8548	1.2396	0.2518	0.7204	0.1128	0.3086	0.0000
	CBTV-1	0.0000	0.0000	0.0000	0.0295	0.0000	0.0000	0.0000	0.0000	0.0000	0.0000	0.0000	0.0000
	CBTA-3	0.4617	0.2567	0.3411	0.0000	0.0087	0.0000	0.0000	0.0000	0.0000	0.0612	0.0935	0.0000
	CBT-3	0.1403	0.4385	0.0731	1.6640	2.6390	0.5477	0.7158	0.0948	0.3649	0.0344	0.0572	0.0000
	CBT-2	0.0000	0.0215	0.0000	0.0000	0.0000	0.0000	0.0607	0.0484	0.0000	0.0000	0.0000	0.0000
	329-11a	0.0000	0.0000	0.0000	0.0000	0.0000	0.0000	0.0554	0.2582	0.0523	0.0276	0.0000	0.0000
	329-11b	0.0000	0.0139	0.0169	0.1524	0.1491	0.2630	0.1943	0.0550	0.1050	0.0000	0.0247	0.0277
	329-11c	0.0000	0.0000	0.0000	0.0170	0.0000	0.0000	0.0737	0.1308	0.0000	0.0330	0.0000	0.0187
	329-11d	0.1285	0.5413	0.1145	0.7637	1.1899	0.3622	0.2969	0.0424	0.0789	0.0190	0.0703	0.0000
	329-11e	0.0000	0.0000	0.0000	0.0554	0.0843	0.0176	0.0368	0.0000	0.0000	0.0000	0.0000	0.0000
	373-12a	0.0000	0.0000	0.0000	0.0000	0.0000	0.0000	0.0000	0.0100	0.0000	0.0455	0.0088	0.0000
	373-12b	0.0234	0.0276	0.0807	0.0000	0.0000	0.0000	0.0000	0.0035	0.0000	0.0413	0.0630	0.0000
	373-12c	0.0297	0.0452	0.0124	0.0000	0.0000	0.0000	0.0000	0.0000	0.0000	0.0000	0.0000	0.0000
	327-13a	0.0000	0.0000	0.0000	0.0000	0.0000	0.0000	0.1053	0.7707	0.0314	0.0000	0.0000	0.0000
	327-13b	0.0000	0.0000	0.0000	0.0000	0.0000	0.0000	0.0157	0.1360	0.0317	0.0146	0.0000	0.0000
	327-13c	0.0000	0.0000	0.0000	0.0000	0.0000	0.0000	0.2999	0.2335	0.1199	0.2178	0.0772	0.0000
	371-14a	0.0000	0.0000	0.0000	0.0000	0.0000	0.0000	0.0000	0.0331	0.0000	0.0341	0.0030	0.0000
	371-14b	0.1078	0.1063	0.0815	0.0000	0.0000	0.0000	0.0000	0.0000	0.0000	0.0046	0.0193	0.0000
	417-15a	0.0467	0.0595	0.0626	0.0000	0.0000	0.0000	0.0000	0.0000	0.0000	0.0000	0.0226	0.0000
	373-15b	0.2426	0.2337	0.0787	0.1663	0.1712	0.1308	0.1767	0.1204	0.1346	0.1241	0.1380	0.0000
	373-15c	0.0000	0.0000	0.0000	0.0000	0.0000	0.0000	0.0413	0.1523	0.0000	0.1298	0.1851	0.0000
	357-16a	0.0143	0.0290	0.0068	0.0000	0.0000	0.0000	0.0000	0.0000	0.0000	0.0352	0.0441	0.0000
	313-16b	0.0000	0.0000	0.0000	0.0103	0.0173	0.0000	0.1367	0.2006	0.1009	0.0917	0.0711	0.0000
	361-17a	0.0000	0.0962	0.0000	0.0236	0.0454	0.0265	0.0000	0.0000	0.0163	0.0000	0.0000	0.0000
	361-17b	0.0000	0.0000	0.0000	0.0256	0.0333	0.0000	0.0154	0.0000	0.0203	0.0000	0.0000	0.0000
	331-18a	0.0000	0.0000	0.0000	0.0396	0.0435	0.0000	1.2613	2.0983	1.0447	0.1981	0.1158	0.0686
	331-18b	1.1828	0.8041	0.3370	0.7599	0.4643	0.3771	0.5084	0.7992	0.2322	0.6126	0.6776	0.7082
	331-18d	0.1087	0.2432	0.1558	0.0392	0.0670	0.0908	0.0543	0.0713	0.0000	0.0810	0.2207	0.0000
	375-19a	0.0087	0.0094	0.0000	0.0000	0.0000	0.0000	0.0000	0.0191	0.0000	0.5218	0.3494	0.0000
	375-19b	0.0209	0.0123	0.0000	0.0000	0.0000	0.0000	0.0000	0.0000	0.0000	0.0000	0.0030	0.0000
	375-19c	0.0234	0.0210	0.0000	0.0000	0.0000	0.0000	0.0000	0.0000	0.0000	0.0042	0.0057	0.0000

C - %w/w
C<0.02
0.02<C<0.1%
0.1%<C<1%
1%<C<10%
10%<C<30%
C>30%

Supplementary Figure 1: Phytocannabinoid analysis of the *Cannabis* extracts. ECI-LC/MS phytocannabinoid profiling of the *Cannabis* extracts which were prepared as described in Materials and Methods in the main text. *Cannabis* extracts were ordered into clusters according to cannabinoid content (% w/w) in each extract with values color-coded according to order of magnitude of concentration. Only cannabinoids identified at a concentration of at least 0.2 (%w/w) in at least one extract were included in this figure.

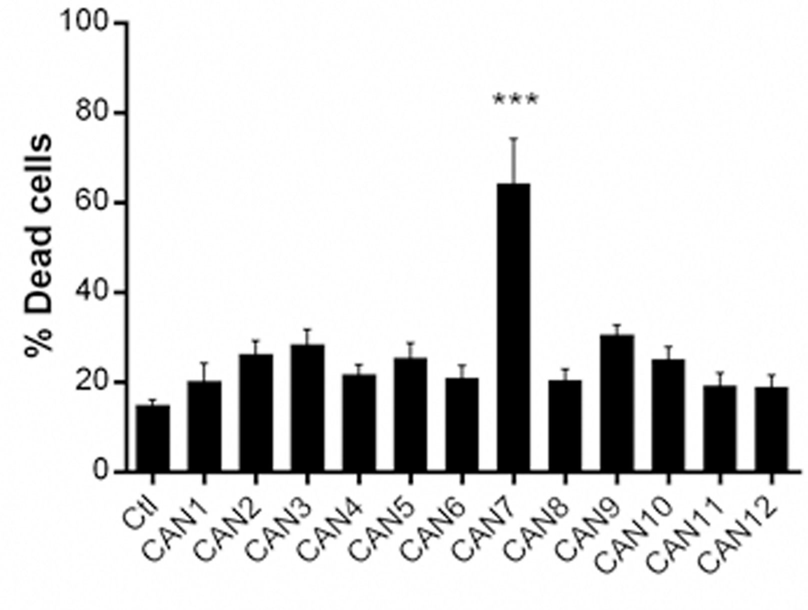

Supplementary Figure 2: The effect of various *Cannabis* extracts on primary airway epithelial cells. Normal primary epithelial cells were incubated for 24 hours incubation with or without (control) 4 µg/ml of CAN1-CAN12. This was followed by the addition of Hoechst and PI and imaged by IXM Micro system, 20× magnification. Live and dead cells were analyzed by the MetaXpress™ software, and data are reported as mean ± SE of % dead cells out of total cells (N=3). Asterisks indicate statistical difference compared to control (*P<0.05, one-way ANOVA).

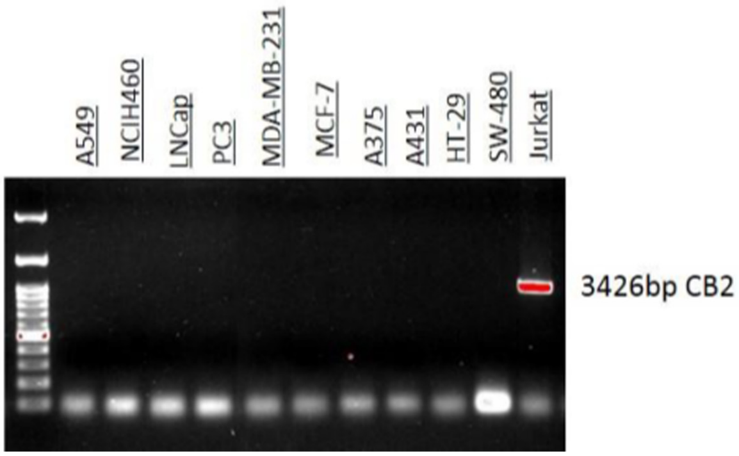

Supplementary Figure 3: Evaluation of CNR2 mRNA expression in ten different cancer cell lines. CNR2 mRNA expression levels were evaluated by PCR, using cDNA. As described in Supplementary Materials.

Type	Cannabinoid	CAN23	CAN19	CAN20	CAN21	CAN24	CAN25	CAN5	CAN13	CAN26	CAN17	CAN14	CAN16	CAN7	CAN18
Δ9-THCtype	THCA	1.1502	1.5803	0.6912	3.5042	4.1435	0.1274	0.3049	5.0325	0.4736	0.0000	0.1255	0.3122	0.0000	0.6908
	THC	46.0497	48.6068	52.1128	52.2002	53.2992	56.1284	56.5847	56.5917	57.3547	60.9241	62.2031	63.1925	67.8032	81.7029
	THCA-C4	0.0000	0.0000	0.0000	0.0128	0.0198	0.0000	0.0000	0.0213	0.0000	0.0000	0.0000	0.0000	0.0000	0.0000
	THC-C4	0.1160	0.1188	0.1696	0.1184	0.1498	0.0000	0.1085	0.1196	0.1278	0.1720	0.2003	0.2159	0.1969	0.1275
	THCVA	0.0213	0.0000	0.0000	0.0452	0.0532	0.0000	0.0000	0.0552	0.0000	0.0000	0.0000	0.0000	0.0000	0.0000
	THCV	0.3933	0.2408	0.4409	0.3724	0.3426	0.2343	0.2072	0.2938	0.3090	0.4601	0.4692	0.4479	0.6137	0.4426
	THCOA	0.0000	0.0000	0.0000	0.0132	0.0134	0.0000	0.0000	0.0255	0.0000	0.0000	0.0000	0.0000	0.0000	0.0000
CBD type	CBDA	0.0917	0.0109	0.0270	0.0546	0.0379	0.0089	0.0000	0.0665	0.0246	0.0000	0.0175	0.0750	0.0000	0.0285
	CBD	0.7699	0.1115	0.1174	0.0846	0.0964	0.1240	0.2125	0.1095	0.1204	0.1325	0.1679	0.3280	0.2107	0.1598
CBG type	CBGA	0.1783	0.1988	0.2964	1.1113	0.4298	0.1016	0.0725	0.2913	0.2919	0.0579	0.0595	0.1588	0.2240	0.4754
	CBG	1.3528	1.7086	1.1200	1.7429	1.1701	0.9061	4.4623	0.8703	1.4233	1.6082	0.6369	1.0870	12.6179	2.2239
	CBGA-C4	0.0116	0.0077	0.0076	0.0151	0.0192	0.0094	0.0408	0.0272	0.0102	0.0000	0.0000	0.0132	0.0362	0.0148
	Sesqui-CBG	0.0246	0.0248	0.0114	0.0154	0.0404	0.0320	0.0310	0.0292	0.0594	0.0280	0.0336	0.0525	0.0160	0.0275
CBC type	CBCA	0.0376	0.0305	0.0466	0.1657	0.0991	0.0273	0.0060	0.1257	0.0378	0.0215	0.0274	0.0435	0.0061	0.0721
	CBC	1.1255	0.8699	0.6844	0.7319	0.7210	0.7091	1.0927	0.4670	0.5878	0.8653	0.6813	0.6439	0.8269	1.0350
CBN type	CBNA	0.0550	0.0334	0.1016	0.1903	0.0788	0.0000	0.0121	0.1660	0.0236	0.0000	0.0296	0.0229	0.0000	0.0617
	CBN	1.6068	0.5138	2.4148	0.4405	0.5195	0.4269	2.7877	0.6746	0.6110	0.5453	1.5265	0.6243	1.0339	1.5517
	CBNV	0.0142	0.0000	0.0207	0.0024	0.0023	0.0000	0.0083	0.0029	0.0000	0.0036	0.0117	0.0043	0.0096	0.0082
	CBL	0.0000	0.0000	0.0000	0.0000	0.0000	0.0000	0.0458	0.0000	0.0000	0.0000	0.0000	0.0000	0.0000	0.0000
CBTtype	CBTA-1	0.0160	0.0194	0.0068	0.0237	0.0406	0.0000	0.0166	0.0340	0.0000	0.0000	0.0000	0.0000	0.0000	0.0050
	CBT-1	2.2479	1.6424	1.7820	2.3609	2.1782	1.1004	4.1060	2.5065	1.2334	0.9925	1.2721	1.3111	3.3258	1.8195
	CBTV-1	0.0175	0.0000	0.0139	0.0152	0.0000	0.0000	0.0000	0.0000	0.0000	0.0000	0.0000	0.0000	0.0295	0.0000
	CBT-3	0.8889	0.7212	1.3833	0.5757	0.4832	0.6269	2.6390	0.6647	0.8046	0.3887	0.5495	0.5503	1.6640	1.9103
	329-11b	0.0875	0.5470	0.1020	0.0522	0.0709	0.0409	0.1491	0.0748	0.0987	0.0358	0.1108	0.3803	0.1524	0.1389
	329-11c	0.0000	0.0909	0.0000	0.0000	0.0000	0.0000	0.0000	0.0000	0.0000	0.0000	0.0000	0.0000	0.0170	0.0000
	329-11d	0.5344	0.4950	0.3515	0.4551	0.4780	0.2922	1.1899	0.3604	0.2622	0.3361	0.4742	0.4629	0.7637	0.6024
	329-11e	0.0400	0.0233	0.0350	0.0235	0.0456	0.0172	0.0843	0.0354	0.0206	0.0069	0.0478	0.0373	0.0554	0.0473
	373-15b	0.2644	0.2457	0.0522	0.3520	0.3424	0.0885	0.1712	0.4375	0.1482	0.1348	0.2806	0.3367	0.1663	0.1918
	373-15c	0.5388	0.1661	0.4819	0.3271	0.1323	0.0000	0.0000	0.2561	0.2482	0.0000	0.1311	0.3167	0.0000	0.0000
	361-17a	0.2379	0.0497	0.2154	0.0819	0.0544	0.0297	0.0454	0.1339	0.0764	0.0000	0.1601	0.0484	0.0236	0.1958
	361-17b	0.0649	0.0211	0.0458	0.0283	0.0256	0.0265	0.0333	0.0511	0.0346	0.0177	0.0404	0.0266	0.0256	0.0764
	331-18a	0.0437	0.0158	0.0192	0.0148	0.0163	0.0215	0.0435	0.0201	0.0212	0.0184	0.0259	0.0267	0.0396	0.0331
	331-18b	0.3667	0.7235	0.2291	0.7731	0.7236	0.3291	0.4643	0.6252	0.6119	0.0000	0.0000	0.8514	0.7599	0.8409
	331-18d	0.0558	0.1570	0.1614	0.0924	0.0000	0.1497	0.0670	0.1388	0.1024	0.0000	0.0349	0.0000	0.0392	0.1833

C - %w/w
C<0.02
0.02<C<0.1%
0.1%<C<1%
1%<C<10%
10%<C<30%
C>30%

Supplementary Figure 4: Phytocannabinoid analysis of the *Cannabis* extracts. ECI-LC/MS phytocannabinoid profiling of 14 Δ9-THC-rich *Cannabis* extracts which were prepared as described in Materials and Methods. *Cannabis* extracts are ordered by increasing content of Δ9-THC (% w/w) in each extract with values color-coded according to concentration order of magnitude of concentration. Only cannabinoids identified at a concentration of at least 0.2 (%w/w) in at least one extract were included in this figure.

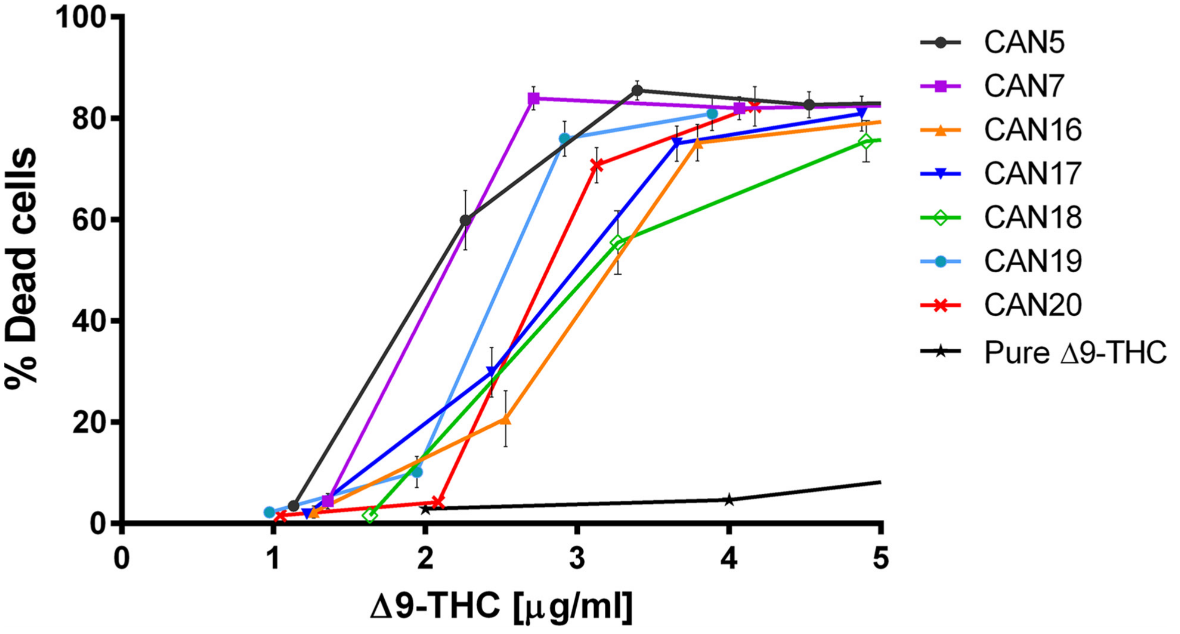

Supplementary Figure 5: The effect of different high Δ9-THC extracts on the survival of A549 cells. A dose-response curve of A549 cells after 24 h incubation with 2-10 μg/ml of seven high-Δ9-THC *Cannabis* extracts or pure Δ9-THC, according to Δ9-THC concentration in each extract. Data are reported as mean ± SE of % dead cells out of total cells (N=5).

Supplementary Table 1: Effect of *Cannabis* extracts on the survival of cancer cell lines

	A549	NCIH460	PC3	LNcAP	HT29	SW480	A431	A375	MDA231	MCF7	U87MG	T98G
**CAN1**	>10	>10	>10	7.91 ± 0.75	>10	>10	>10	>10	>10	>10	>10	>10
**CAN2**	3.89 ± 0.17	4.61 ± 0.57	6.07 ± 0.36	5.99 ± 0.19	>10	3.92 ± 0.36	6.26 ± 0.33	4.75 ± 0.16	3.57 ± 0.36	7.04 ± 0.31	3.32 ± 0.14	3.18 ± 0.25
**CAN3**	7.43 ± 0.15	7.88 ± 0.56	9.39 ± 0.22	>10	>10	6.33 ± 0.39	9.16 ± 0.44	7.82 ± 0.30	5.74 ± 0.47	>10	5.94 ± 0.20	5.12 ± 0.21
**CAN4**	5.84 ± 0.20	6.59 ±0 .39	7.86 ± 0.22	4.44 ± 0.35	>10	6.16 ± 0.50	8.92 ± 0.39	6.75 ± 0.20	7.35 ± 0.52	>10	7.55 ± 0.42	6.69 ± 0.28
**CAN5**	3.65 ± 0.19	4.68 ± 0.77	5.94 ± 0.22	6.22 ± 0.27	>10	3.70 ± 0.39	5.90 ± 0.45	4.97 ± 0.21	4.54 ± 0.17	6.21 ± 0.23	4.16 ± 0.13	3.53 ± 0.20
**CAN6**	8.30 ± 0.21	7.70 ± 0.63	9.14 ± 0.18	4.29 ± 0.35	>10	5.75 ± 0.43	>10	7.92 ± 0.34	8.35 ± 0.55	>10	>10	8.54 ± 0.41
**CAN7**	3.06 ± 0.18	4.11 ± 0.81	3.85 ± 0.41	5.21 ± 0.30	>10	3.71 ± 0.38	5.74 ± 0.42	4.51 ± 0.33	3.24 ± 0.19	5.11 ± 0.21	2.97 ± 0.13	3.07 ± 0.17
**CAN8**	>10	>10	>10	7.34 ± 0.60	>10	>10	>10	>10	>10	>10	>10	>10
**CAN9**	4.90 ± 0.14	5.17 ± 0.55	6.36 ± 0.28	7.77 ± 0.42	>10	4.42 ± 0.30	6.99 ± 0.30	4.92 ± 0.20	3.90± 0.31	8.11 ± 0.47	3.71 ± 0.15	3.25 ± 0.17
**CAN10**	4.33 ± 0.15	4.92 ± 0.42	5.69 ± 0.33	6.03 ± 0.20	>10	4.81 ± 0.53	9.96 ± 0.58	4.97 ± 0.36	3.77±0.27	7.82 ± 0.59	3.64 ± 0.13	3.15 ± 0.22
**CAN11**	>10	>10	>10	7.39 ± 0.53	>10	>10	>10	>10	>10	>10	>10	>10
**CAN12**	6.85 ± 0.15	8.04 ± 0.48	8.92 ± 0.11	>10	>10	7.01 ± 0.48	9.23 ± 0.48	8.35 ± 0.31	8.64 ± 1.13	>10	6.39 ± 0.12	6.40 ± 0.16
**Δ^9-THC**	7.37 ± 0.18	7.7 3 ± 1.02	>10	>10	>10	9.94 ± 0.82	9.41 ± 1.14	9. 23± 0.96	7.24 ± 0.32	>10	6.08 ± 0.29	6.23 ± 0.35

Various cancer cell lines were treated with (2-10 µg/ml) concentrations of *Cannabis* extracts and 2-10 µg/ml of pure Δ^9-THC for 24 h. Cells were imaged via ImageXpress Micro XLS (20× magnification) after adding Hoechst and PI dyes to differentiate between live and dead cells. The number of live and dead cells were analyzed by MetaXpress® software. Data are reported as mean ± S.E. of LC50 values (µg/ml) (n ≥ 5).

Supplementary Table 2: Correlation between phytocannabinoid content and LC50 values of 14 Δ^9-THC-rich extracts on the A549 cell line

Cannabinoid	r	p-value	Adjusted p-value
**CBG**	0.6664	0.0002	0.0031
**CBT-3**	0.4621	0.0045	0.0582
**THC**	0.4553	0.0049	0.0582
**329-11d**	0.4346	0.0062	0.0677
**373-15c**	0.3237	0.0198	0.1976
**CBC**	0.2995	0.0250	0.2246
**CBT-1**	0.2151	0.0540	0.4316
**THCA**	0.1805	0.0729	0.5103
**THCV**	0.1335	0.1087	0.6521
**CBN**	0.0712	0.1831	0.9154
**331-18b**	-0.0383	0.4847	1.0000
**CBD**	-0.0416	0.5011	1.0000
**CBGA**	-0.0689	0.6940	1.0000
**329-11b**	-0.0798	0.8470	1.0000

r- Pearson correlation coefficient, adjusted p-value is according to the Holm-Bonferroni correction method for multiple tests.
